# Comparing tuberculosis gene signatures in malnourished individuals using the TBSignatureProfiler

**DOI:** 10.1186/s12879-020-05598-z

**Published:** 2021-01-22

**Authors:** W. Evan Johnson, Aubrey Odom, Chelsie Cintron, Mutharaj Muthaiah, Selby Knudsen, Noyal Joseph, Senbagavalli Babu, Subitha Lakshminarayanan, David F. Jenkins, Yue Zhao, Ethel Nankya, C. Robert Horsburgh, Gautam Roy, Jerrold Ellner, Sonali Sarkar, Padmini Salgame, Natasha S. Hochberg

**Affiliations:** 1 Division of Computational Biomedicine, Boston University School of Medicine, Boston, MA USA; 2 Bioinformatics Program, Boston University, Boston, MA USA; 3 Division of Computational Biomedicine and Bioinformatics Program, Boston University, Boston, MA USA; 4 Boston Medical Center, Boston, MA USA; 5 Government Hospital for Chest Diseases, Puducherry, India; 6 Jawaharlal Institute of Postgraduate Medical Education and Research, Puducherry, India; 7 Department of Epidemiology, Boston University School of Public Health, Boston, MA USA; 8 Department of Medicine, Center for Emerging Pathogens, Rutgers New Jersey Medical School, Newark, NJ USA; 9 Section of Infectious Diseases, Boston University School of Medicine, Boston, MA USA

**Keywords:** Tuberculosis, RNA-sequencing, Gene sets, Signatures, Biomarkers, Latent tuberculosis infection, Malnutrition

## Abstract

**Background:**

Gene expression signatures have been used as biomarkers of tuberculosis (TB) risk and outcomes. Platforms are needed to simplify access to these signatures and determine their validity in the setting of comorbidities. We developed a computational profiling platform of TB signature gene sets and characterized the diagnostic ability of existing signature gene sets to differentiate active TB from LTBI in the setting of malnutrition.

**Methods:**

We curated 45 existing TB-related signature gene sets and developed our TBSignatureProfiler software toolkit that estimates gene set activity using multiple enrichment methods and allows visualization of single- and multi-pathway results. The TBSignatureProfiler software is available through Bioconductor and on GitHub. For evaluation in malnutrition, we used whole blood gene expression profiling from 23 severely malnourished Indian individuals with TB and 15 severely malnourished household contacts with latent TB infection (LTBI). Severe malnutrition was defined as body mass index (BMI) < 16 kg/m2 in adults and based on weight-for-height Z scores in children < 18 years. Gene expression was measured using RNA-sequencing.

**Results:**

The comparison and visualization functions from the TBSignatureProfiler showed that TB gene sets performed well in malnourished individuals; 40 gene sets had statistically significant discriminative power for differentiating TB from LTBI, with area under the curve ranging from 0.662–0.989. Three gene sets were not significantly predictive.

**Conclusion:**

Our TBSignatureProfiler is a highly effective and user-friendly platform for applying and comparing published TB signature gene sets. Using this platform, we found that existing gene sets for TB function effectively in the setting of malnutrition, although differences in gene set applicability exist. RNA-sequencing gene sets should consider comorbidities and potential effects on diagnostic performance.

**Supplementary Information:**

The online version contains supplementary material available at 10.1186/s12879-020-05598-z.

## Background

Tuberculosis (TB) is the leading cause of death due to an infectious disease worldwide, killing 1.6 million people in 2017 [1]. The EndTB strategy aims to reduce TB deaths by 95% and to cut new cases by 90% between 2015 and 2035 [2]. A critical component of this strategy is early identification of individuals with TB and prevention of transmission. Although the roll-out of GeneXpert has facilitated rapid TB diagnosis, the test has limitations (e.g., lower sensitivity if low bacillary burden, in children, and in extra-pulmonary disease) [3–5]. Furthermore, not all individuals with possible pulmonary TB are able to produce sputum [6]. Newer blood-based diagnostics using gene expression profiles have the potential to address the limitations of GeneXpert and other sputum-based tests [7].

Over the past several years, researchers have been able to identify nearly four dozen gene expression signatures that distinguish TB disease from latent TB infection (LTBI) [8, 9], TB from other infections [10–12], incipient pre-symptomatic TB disease and/or the future development of TB disease in those with LTBI [13–15], and response to therapy [16, 17]. Signatures can be used to understand the heterogeneous response to TB and help identify the pathways and underlying biology of TB disease progression. These signatures have been developed using multiple profiling technologies (microarray, RNA-sequencing, rt-PCR) and using a diverse set of computational and machine learning prediction algorithms. Some of these signatures were developed using direct training or cross-validation approaches on a single study, while others were developed using a meta-analytical approach [17, 18]. Furthermore, several of these gene signatures have been validated by independent research teams on diverse cohorts in different settings and using multiple computational algorithms [19–21]. Importantly, recent studies have systematically compared the performance of TB signatures, and their associated gene sets and original predictive models, across a multiple of TB datasets to compare the performance of these signatures to predict TB outcomes [20, 21]. However, despite this work, there is not a single resource of compiled signature gene lists, methods or biomarkers to apply to new datasets, and most gene sets have not been independently validated using alternative computational methodologies.

Existing studies of blood-based TB diagnostics have another important limitation: most have not evaluated the impact of comorbidities on the modulation of the TB signature. In high-TB burden settings, much of the population has comorbidities that affect host immune response, and likely alter gene signatures of TB disease. Some of these have been directly studied (e.g., diabetes, HIV) [22–24] and others have not (e.g., malnutrition, pregnancy, parasites). In particular, the role of malnutrition, which is known to modulate the innate and adaptive immune responses, has not been explored [25, 26]. Malnutrition affects much of the population in TB endemic countries including one-third of the adult population in India, the country with 27% of the world’s TB cases [1]. It is the most common secondary immunodeficiency and has been termed nutritional acquired immunodeficiency syndrome [27, 28]. Undernutrition appears to impact both the innate and adaptive immune systems [29], and so can conceivably alter gene expression in these patients in significant ways. For example, undernourished individuals have been noted to have decreased expression of Th1 cytokines and increased concentrations of Th2 cytokines, which hobbles the Th1 response against Mtb [30, 31]. Prior research has also suggested that undernutrition may also diminish the effectiveness of TB vaccines. Furthermore, a study over two decades in the United States found that a BMI < 18.5 kg/m2 was associated with an adjusted hazard ratio of 12.43 (CI: 95% CI: 5.75, 26.95) for developing TB disease as compared to those with BMI greater than 18.5. In India, more than 50% of TB cases are attributable to undernutrition in most states [32]. Because of the significant TB risk malnutrition poses and the gap in current knowledge, we sought to determine whether the published gene lists indicating TB disease accurately discriminate TB from LTBI in the setting of malnutrition in India.

In this work, we curated almost four dozen existing TB-related signature gene sets and developed our TBSignatureProfiler software toolkit. We also added two single-gene biomarkers to this comparison that were compared in a previous meta-analysis [21]. This platform was used to evaluate the function of these signatures for distinguishing between TB and LTBI in severely malnourished individuals. We applied the TBSignatureProfiler to this condition to determine whether existing TB gene sets work in a severely malnourished population. While it is unlikely that these signatures will be implemented in clinical practice for detecting TB disease, we do note that many existing signatures were developed for this purpose. Thus, comparisons between prevalent and latent TB is the logical first step in evaluating and validating these signature gene sets in the setting of malnutrition. Once these signatures are established and validated, they can be used for more innovative and useful applications, such as predicting risk of progression or worsening disease, monitoring treatment efficacy, or the diagnosis of extrapulmonary disease.

## Methods

### Collection of published TB signature gene sets

Our goal was to compile a comprehensive set of multi-gene signature gene lists and make them available through our TBSignatureProfiler platform. The only criteria for inclusion in this study was that the signature gene set consisted of at least two genes and was used and presented as a biomarker of a TB outcome (disease, risk, treatment, etc) in a peer reviewed publication. We collected a set of 45 previously published gene sets in total (Table 1). References for these gene sets are available in the Supplementary Materials and from the TBSignatureProfiler software documentation. These gene sets were derived from multiple studies, using several transcriptional profiling platforms, and using disparate predictive methods and algorithms. As such, we defined the term “gene sets” or “signature” as the collection of genes that were used in the predictive model in its original study. We then define the “gene set/signature strength” or “gene set/signature score” by the single sample gene set enrichment score for that set. For presentation gene signatures are labeled using the first author’s last name and the number of genes in the signature (e.g., Berry_393). Gene sets that focus on the presence of comorbidity with TB and another disease have additional labels. Details for these naming conventions are available in the Supplementary Methods. We also included two previously proposed single gene biomarkers, NPC2 [33] and BATF2 [34, 35], using their gene expression counts in our comparison.

**Table 1:** Details for gene signatures curated and available in the TBSignatureProfiler. References for these signatures are available in the (Additional file 1) and from the TBSignatureProfiler software documentation (?TBSignatures)

	Differential expression		Area under the ROC curve				
Signature	*P*-value	-10 Log10 P	LowerAUC	AUC	UpperAUC	LowerCISunXu	UpperCISunXu
**Bloom_OD_144**	0	212.4	0.961	0.989	1	0.967	1
**Thompson_9**	0	198	0.954	0.983	1	0.948	1
**NPC2**	0	214	0.9417	0.98	1	0.947	1
**Blankley_5**	0	225.3	0.942	0.977	1	0.931	1
**Tornheim_RES_25**	0	181.1	0.93	0.972	1	0.926	1
**Roe_OD_4**	0	198.3	0.919	0.969	1	0.916	1
**Gjoen_7**	0	114.8	0.911	0.966	1	0.917	1
**Kaforou_27**	0	178.7	0.908	0.966	0.995	0.915	1
**Tornheim_71**	0	193.5	0.921	0.966	0.997	0.919	1
**Blankley_380**	0	154.7	0.921	0.963	0.997	0.913	1
**Sambarey_HIV_10**	0	172.4	0.897	0.96	1	0.905	1
**Walter_51**	0	138.5	0.905	0.957	0.991	0.903	1
**Jacobsen_3**	0	158	0.872	0.946	0.991	0.881	1
**Gliddon_OD_3**	0	154.9	0.875	0.943	0.986	0.873	1
**Zak_RISK_16**	0	128.3	0.868	0.94	0.989	0.872	1
**Sweeney_OD_3**	0	116.4	0.849	0.938	0.983	0.868	1
**BATF2**	0	138	0.863	0.935	0.986	0.863	1
**Roe_3**	0	112.3	0.84	0.929	0.988	0.847	1
**Esmail_203**	0	125	0.861	0.926	0.983	0.849	1
**Kaforou_OD_53**	0	122.7	0.826	0.921	0.978	0.831	1
**Rajan_HIV_5**	0	118.4	0.842	0.906	0.986	0.81	1
**Esmail_82**	0	111.7	0.797	0.895	0.972	0.781	1
**Berry_393**	0	114.2	0.812	0.886	0.958	0.784	0.989
**Anderson_OD_51**	0	101.8	0.797	0.884	0.962	0.779	0.988
**Huang_OD_13**	0	106.9	0.778	0.884	0.964	0.775	0.992
**Maertzdorf_OD_100**	0	112.7	0.785	0.872	0.956	0.756	0.988
**Singhania_OD_20**	0.0001	93.4	0.755	0.861	0.958	0.737	0.985
**Kaforou_OD_44**	0.0001	90.8	0.76	0.852	0.937	0.734	0.971
**Gjoen_10**	0.0001	93.8	0.747	0.849	0.943	0.729	0.97
**Verhagen_10**	0.0001	94	0.747	0.844	0.921	0.722	0.965
**Gliddon_OD_4**	0	103.8	0.739	0.838	0.926	0.714	0.962
**Jenum_8**	0.0001	90.4	0.724	0.838	0.936	0.712	0.964
**Esmail_OD_893**	0.0002	83.8	0.719	0.835	0.919	0.704	0.966
**Suliman_4**	0.0002	86.9	0.689	0.83	0.917	0.697	0.962
**Berry_OD_86**	0.0003	82.1	0.716	0.821	0.941	0.686	0.956
**Thompson_RES_5**	0.0005	76.2	0.696	0.798	0.911	0.656	0.941
**Thompson_FAIL_13**	0.0008	71.3	0.698	0.787	0.89	0.642	0.932
**Walter_PNA_47**	0.0046	53.8	0.643	0.773	0.897	0.624	0.922
**Walter_PNA_119**	0.002	62.1	0.643	0.77	0.902	0.602	0.938
**Leong_24**	0.0115	44.6	0.576	0.73	0.843	0.564	0.896
**Leong_RISK_29**	0.0315	34.6	0.589	0.716	0.857	0.546	0.886
**Anderson_42**	0.324	11.3	0.522	0.662	0.78	0.482	0.853
**Suliman_RISK_4**	0.024	37.3	0.538	0.662	0.814	0.486	0.842
**Qian_OD_17**	0.1615	18.2	0.514	0.611	0.757	0.421	0.801
**Sloot_HIV_2**	0.1	23	0.515	0.605	0.744	0.421	0.789
**Maertzdorf_4**	0.8307	1.9	0.515	0.58	0.757	0.376	0.783
**Lee_4**	0.3019	12	0.511	0.511	0.695	0.322	0.678

### TBSignatureProfiler platform

The 45 previously published gene sets of TB outcomes are included in our TBSignatureProfiler, a novel R package that allows users to quickly and easily perform single sample pathway enrichment analysis using our set of TB signature gene sets and multiple scoring methods, including ssGSEA, GSVA, PLAGE, combining Z-scores, ASSIGN and singscore [36–41] (these methods are detailed in the Supplementary Methods). This workflow can then be used for profiling and visualizing these gene sets/pathways and plotting functions in our TBSignatureProfiler R package. The R package is available on GitHub (https://github.com/compbiomed/TBSignatureProfiler) and through Bioconductor (https://bioconductor.org/packages/release/bioc/html/TBSignatureProfiler.html). Additional details for the scoring visualization, and comparison functions are detailed in the Supplementary Methods and in the software package vignette.

### Malnourished individuals from RePORT-India

Our malnourished samples came from the Regional Prospective Observational Research in TB (RePORT)-India cohort based at Jawaharlal Institute of Postgraduate Medical Education and Research (JIPMER). The study is conducted in collaboration with Boston Medical Center and Rutgers-New Jersey Medical School. Ethical approval was obtained by the JSAC and IEC committees of JIPMER and the institutional review boards of Boston Medical Center and Rutgers University. This household contact study enrolls newly-diagnosed smear-positive, culture-confirmed pulmonary TB cases identified at Revised National TB Control Programme clinics as well as their household contacts; additional study details have been previously reported [19, 42–44]. In brief, index cases are visited at enrollment, 1, 2, 6 and 12 months and household contacts at enrollment, 12 and 24 months. Blood is collected in PaxGene RNA sequencing tubes at each time point. Household contacts undergo tuberculin skin testing (TST) for LTBI and are monitored for symptoms of active TB; sputum testing is done on symptomatic individuals.

In addition to demographic characteristics, questionnaires address relevant comorbidities that affect host response and TB risk including HIV, diabetes, renal failure, other immunosuppressive conditions, alcohol use (and at-risk alcohol use based on the Alcohol Use Disorders Identification Test [45], tobacco use, and other parameters. These values are summarized in Table 2. Participant BMI is measured at baseline and categorized into severe malnutrition (BMI < 16 kg/m2), malnutrition (16–18.4), and normal/overweight (> 18.4) henceforth referred to as well-nourished. In individuals less than 18 years of age, BMI was categorized based on standard deviations relative to the World Health Organization median: children whose BMI was more than two standard deviations away from the median for their age were categorized as malnourished [46]. In index cases, blood samples are taken to diagnose diabetes mellitus (random blood sugar > 200 mg/dL) and HIV.

**Table 2:** Demographic characteristics of new smear-positive pulmonary tuberculosis patients and household contacts, India (*n* = 38)

	Malnourished Index Case (***n***=23)	Malnourished Household Contact (***n***=15)	***P***-value
**RNA-seq Processing Batch**			
Batch 1	8	1	
Batch 2	15	14	
**Demographic Characteristics**			
Male, n(%)	16 (69.6)	7 (46.7)	0.1903
Age, median (range)	47 (15-67)	13 (10-23)	<0.0001
Any alcohol use, n (%)	14 (60.9)	0	<0.0001
Risky alcohol use, n (%)	10 (43.5)	0	<0.0001
Ever a smoker, n (%)	15 (56.5)	0	<0.0001
BMI, median (range)	14.8 (11.5-15.9)	15.1 (13.7-15.8)	0.2221
**Clinical Characteristics**			
AFB smear grade, n(%)			
1+	10 (43.5)	--	
2+	5 (21.7)	--	
3+	8 (34.8)	--	
MGIT time to positive, median (range)	6 (3-14)	--	
Duration of cough before treatment, median (range)	4 (2-5)	--	
Tuberculin Skin Test Millimeters of Induration, median (range)	--	7 (5-15)	

### RNA-sequencing data generation and processing

We analyzed RNA-seq data from enrollment PaxGene tubes from a subset of 23 severely malnourished individuals with TB and 15 severely malnourished tuberculin skin test positive (TST ≥5 mm) household contacts as previously described [19]. The data were batch corrected using ComBat-Seq [47, 48] (Supplementary Figure 1). Differential expression between TB and LTBI samples produced 6706 differentially expressed features using an adjusted *p*-value (FDR) cutoff of 0.01, including 4913 protein coding genes, 1052 lncRNAs, 135 T cell receptive elements, 19 immunoglobulin genes, and 13 miRNAs. The list of protein coding genes was used to develop a list of differentially expressed genes and pathways of TB vs LTBI. Detailed methods for the processing of the PaxGene tubes, RNA-sequencing, and data analysis are available in the Supplementary Methods.

## Results

### Analysis and overlap of existing TB signature gene sets

**Fig. 1:**
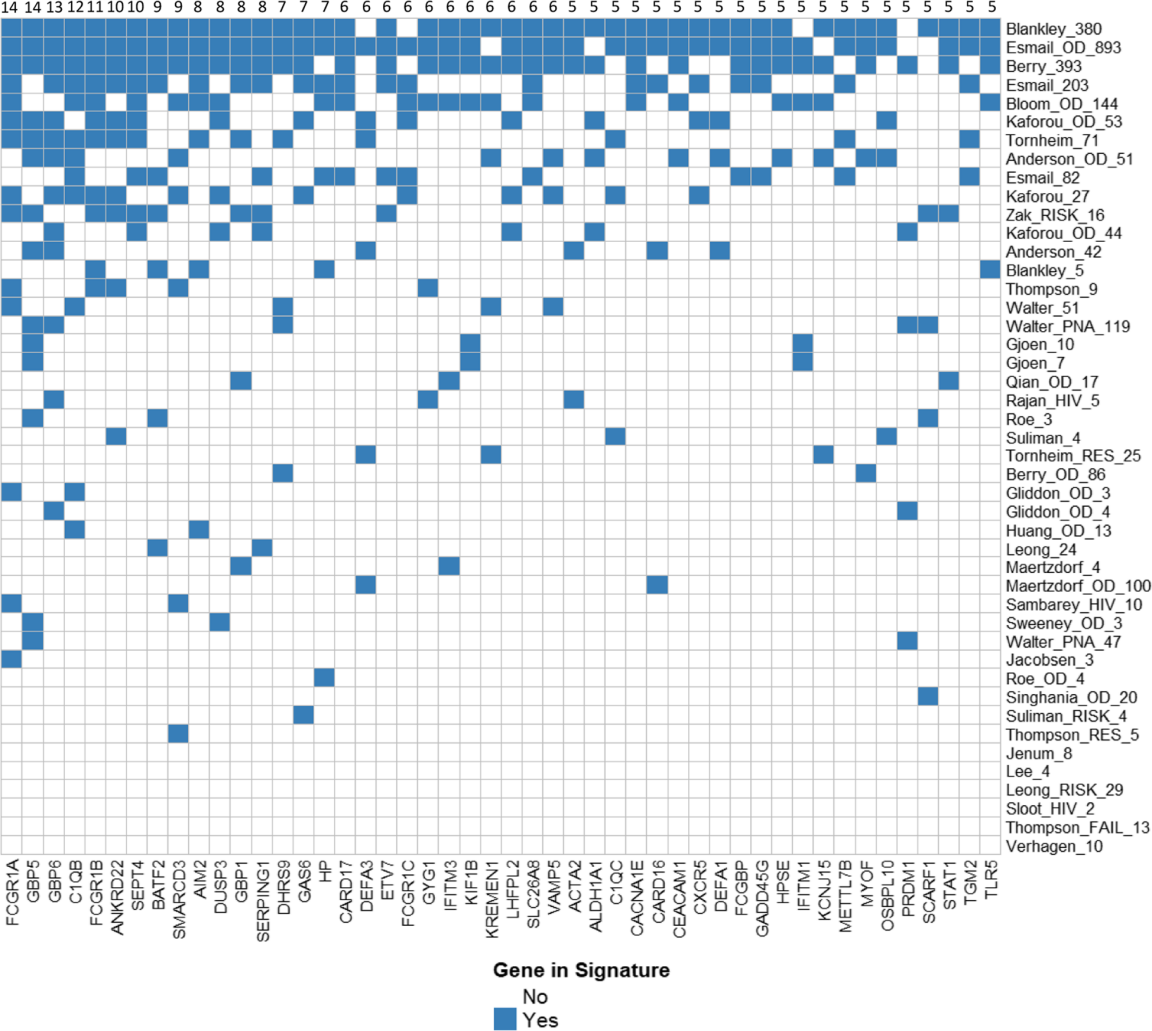
Overlap of genes in the TB signature cohort listed in 5 or more signatures. Of the 1513 unique genes in the 45 signatures, 48 are listed in 5 or more signatures. Most of these signature genes are contained in the large Esmail_893 gene, Berry_393 gene, and Blankley_380 gene signatures

The 45 TB signature gene sets described in Table 1 consist of between 2 and 700 unique UCSC gene symbol annotations. Overall, these gene sets include 1513 unique TB associated genes. Most genes (965, 63.8%) are listed in a single gene set and 96.8% (1465 genes) are listed in four or fewer gene sets; only 48 (3.2%) genes are listed in five or more gene sets (Fig. 1). Individual genes that occur frequently include FCGR1A, GBP5, GBP6, C1QB, FCGR1B, SEPT4, and ANDKRD22 (in 14, 14, 13, 12, 11, 11, and 10 of the signature gene sets, respectively). Our previously evaluated biomarkers, NPC2 and BATF2 appeared in 1 and 9 signature gene sets, respectively. Additional file 4 provides a matrix of overlap between the individual multi-gene sets. While some smaller gene sets significantly overlap with larger ones (e.g. 15/16 genes from Zak_RISK_16 are present in Berry_393), most gene sets were largely non-overlapping from the single gene perspective. However, despite the small number of overlapping individual genes, there are many common functional families that are represented across multiple gene sets. Most of these are associated with host inflammatory response and immune regulation (see Discussion section).

### Demographics of malnourished TB cases and controls

(Table 2). Overall, 16/23 (69.6%) of the individuals with TB were male compared to 7/15 (46.7%) of those with LTBI (*p*-value = 0.19). The median age of those with TB was 47 years (range 15–67), compared to 13 (range 10–23) for those with LTBI (*p*-value< 0.001). There were 14 (60.9%) of those with TB who reported drinking alcohol, of which 10 (43.5%) reported at-risk alcohol use. There was no alcohol or tobacco use reported by those with LTBI. None of the participants had HIV infection.

### Analysis of TB signatures in malnourished individuals

#### Differential gene/pathway expression

We found 4913 significant differentially expressed protein coding genes using an FDR threshold of 0.01; 56.9% of the genes from the 45 TB signature gene sets were present in that list. A pathway enrichment analysis using the 1000 most significant genes resulted in multiple relevant enriched pathways, including the NF-kappa B signaling pathway, cytokine-cytokine receptor interaction, and multiple infection response pathways (including response to TB). We used the 500 most differentially expressed genes to create an unsupervised, clustered heatmap (Supplementary Figure 2), which separated the majority of those with TB from those without.

**Fig. 2:**
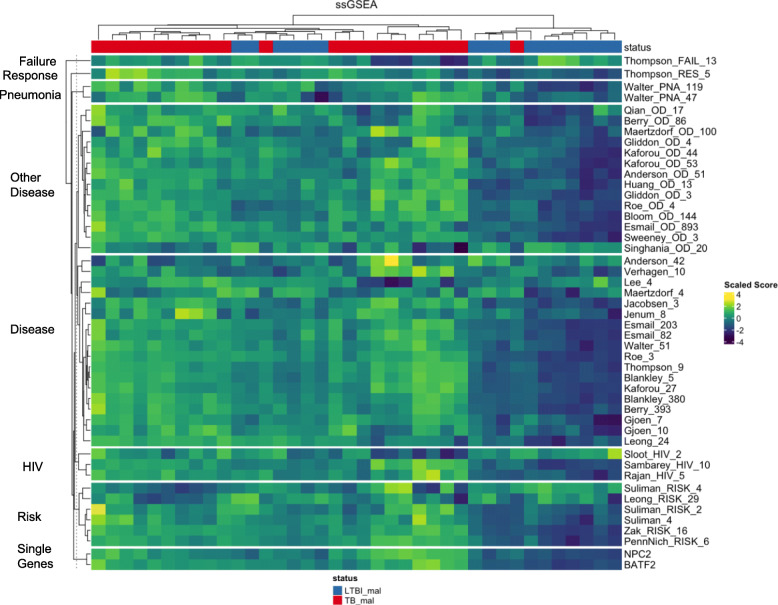
A heatmap displaying the scaled ssGSEA scores for all 45 gene sets (rows) for the samples of malnourished TB and LTBI individuals (columns). Higher scores trend towards yellow-green and lower scores trend towards blue-purple. The color bar at the top designates whether the sample is from an LTBI individual (blue) or an individual with active TB (red). These signatures are able to separate most of the TB samples from the LTBI samples. The pathway signature scores are largely concordant. This heatmap was generated using the SignatureHeatmap() function from the TBSignatureProfiler

We applied the TBSignatureProfiler to evaluate the performance of existing TB signature gene sets on our data. We used the SignatureHeatmap() function with ssGSEA scoring to evaluate the scores for all 45 gene sets simultaneously (Fig. 2). Similar plots using the GSVA and PLAGE scores are available in Supplementary Figures 3 and 4. The heatmap illustrates that the scores are highly concordant across samples and that the signature gene sets are able to classify TB from LTBI. Specifically, the top four clusters segregated by the dendrogram consisted of one cluster with generally low gene set scores comprising only LTBI samples (*n* = 9), two clusters with the highest gene set scores for most pathways consisting of only TB samples (*n* = 11 and 8), and a fourth cluster consisting of mild to moderate scores for most pathways that consisted of both LTBI (*n* = 7) and TB samples (*n* = 3). Despite general agreement, it is clear that the signature gene sets are not completely concordant, and that all the signature gene sets provide more classification accuracy than each individual signature gene set.

#### Evaluation of individual signature gene set performance

**Fig. 3:**
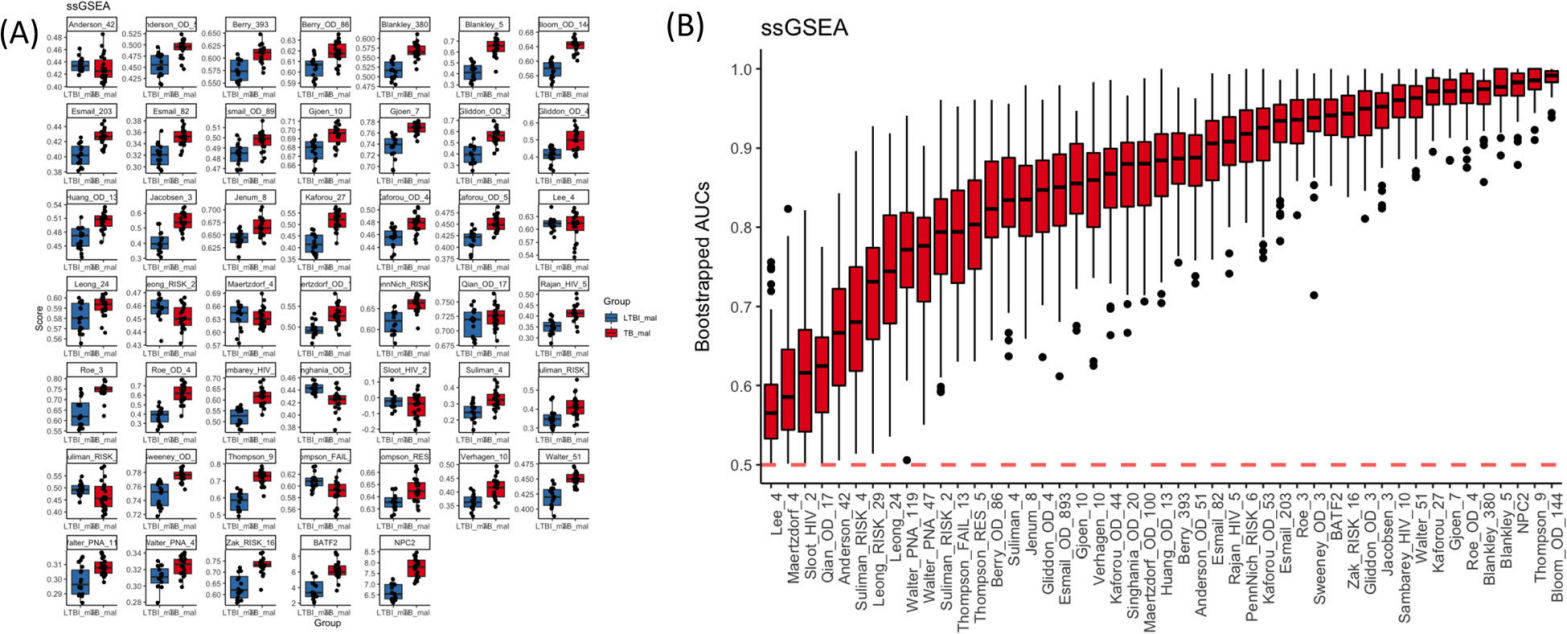
**a** Boxplots of the ssGSEA scores for each signature individually show that some of the signatures are highly predictive of TB compared to LTBI in malnourished individuals. **b** Boxplots for the AUCs (y-axis) from bootstrapped samples for each pathway (x-axis) demonstrate that that most of the signatures were able to classify TB from LTBI, although some of the signatures there of the signatures, including Maertzdorf _4, Lee_4, and Sloot_HIV_2, had boxplots arms below the 0.5 mark. These figures were generated using the SignatureBoxplot() and AUCBoxplot() functions of the TBSignatureProfiler

The performance of signature gene sets can be evaluated using boxplots of individual gene set scores. We used the signatureBoxplot() function to generate a matrix of boxplots for ssGSEA (Fig. 3a), GSVA (Supplementary Figure 5), and PLAGE (Supplementary Figure 6) scores for each gene set. Each pair of boxplots compares the individual signature gene set scores for the TB (red) vs LTBI (blue) samples. The tableAUC() and compareBoxplots() functions evaluated the predictive accuracy and compared gene sets (Fig. 3b and Supplementary Table 1). These boxplots and table values are generated by bootstrapping gene set scores and calculating the AUC of the ROC plot. The ROC curves for these were also generated using the signatureROCplot_CI() function (Supplementary Figure 7). The bootstrapped confidence intervals were supplemented with more direct intervals using AUC variance and estimation procedures defined previously [49, 50]. We note that most of the signatures in our malnutrition data, the bootstrapped confidence intervals were more conservative than the more direct approach. These results show that most gene sets are able to distinguish between the TB and LTBI samples. The Sambarey_HIV_10 (AUC = 0.960) and Thompson_9 (AUC = 0.983) gene sets were two of the best performing gene sets in distinguishing LTBI from TB disease among severely malnourished individuals. Other gene sets also performed extremely well (AUCs> 0.935), including Sweeney_OD_3 gene set (AUC = 0.938), which is being pushed forward as a PCR-based diagnostic in the field. The single gene biomarkers also had very high sensitivity: NPC2 (AUC = 0.980, rank 3/47) and BATF2 (AUC = 0.935, rank 15/47). However, there were a few gene sets that did not perform well in classifying TB from LTBI: Anderson_42 (AUC = 0.662; *p*-value = 0.32), Qian_OD_17 (AUC = 0.611; *p*-value = 0.16), Sloot_HIV_2 (AUC = 0.605; *p*-value 0.10), Maertzdorf_4 (AUC = 0.580; *p*-value = 0.83), and Lee_4 (AUC = 0.511; *p*-value = 0.30) had t-test *p*-values above 0.1 using ssGSEA scores.

#### Evaluation of gene set enrichment scoring methods

**Fig. 4:**
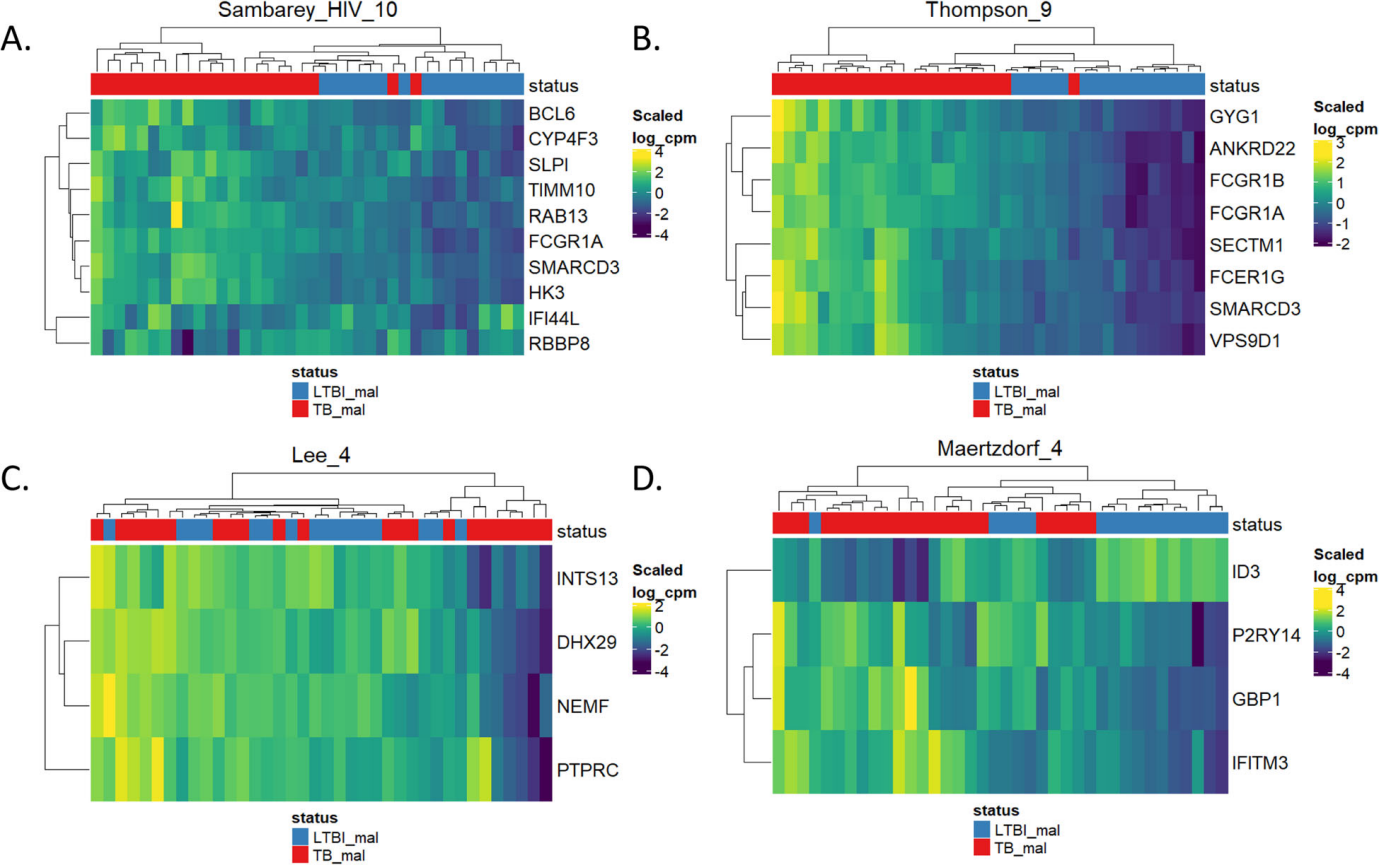
Heatmaps for the gene-level (rows) data for the TB and LTBI samples (columns) for the (**a**) Sambarey_HIV_10, (**b**) Thompson_9, (**c**) Lee_4, and (**d**) Maertzdorf_4 signature gene sets. The Sambarey_HIV_10 and Thompson_9 signatures scored well with the ssGSEA algorithm scoring, whereas the Lee_4 and Maertzdorf_4 gene sets scored poorly. These heatmaps were generated using the signatureGeneHeatmap() function from the TBSigntureProfiler

We used the signatureGeneHeatmap() function to evaluate the gene-level performance of a few pathways one at a time (Fig. 4). The genes in Samabrey_HIV_10 and Thompson_9 segregate malnourished TB and LTBI. The Lee_4 gene set showed poor performance using this metric, as there is no clear clustering of genes and an up-regulation of the four genes among both TB and LTBI. The Maertzdorf_4 gene set showed better performance in clustering and visual analysis than the ssGSEA and AUC analyses showed. This gene set performed better with GSVA (AUC = 0.764) and PLAGE (AUC = 0.932). This is likely because ssGSEA scoring (and GSVA to a lesser extent) relies heavily on concordance of genes (i.e., all are either up-regulated or down-regulated), and thus a gene set such as Maertzdorf_4 that consists of genes that are negatively correlated or pick out different features from samples do not score well with this algorithm. Conversely, we observed that Thompson_9, which consists of highly concordant/redundant genes, had among the best AUCs with ssGSEA (AUC = 0.983), GSVA (AUC = 0.980), and PLAGE (AUC = 0.983).

## Discussion

In this study, we present our set of 45 curated TB signature gene sets along with our TBSignatureProfiler software and use it to assess the impact of malnutrition on discriminative ability of a large number of signature gene sets. The TBSignatureProfiler is an important contribution that provides the first comprehensive, open-source evaluation tool to compare TB signature gene sets in a direct and reproducible way. This automated platform enables investigators to apply nearly three dozen TB gene sets directly to their datasets using multiple different scoring methods with tools to visualize signature gene set strength. Future analyses performed using these same gene sets on additional datasets can be directly compared with past results using the same scoring methods and analytic approach. In addition, new/future signature gene sets can be added and evaluated in a simple and straight-forward way—by merely adding them to the TB signature gene sets collection in the software. This functionality has never been previously available in the TB research field, despite the publication of many dozens of previous gene expression studies, signatures, previous evaluations and metanalyses [17, 19, 20]. Ultimately, the TBSignatureProfiler will enable investigations into whether signature gene sets work in different geographic settings and in the context of different social conditions, contexts, or co-morbidities (e.g., high alcohol use), and efficiently evaluate and compare new signature gene sets in these populations as they are developed.

Overall, there were very few genes that overlapped between the signature gene sets. There were, however, many common functional families that are represented across the gene sets. For example, guanylate-binding proteins (GBPs) are IFN-induced GTPases and contribute to an inflammatory response by activating the NLRP3 and AIM2 inflammasome assembly [51–53]. Interferons are produced during Mtb infection which could lead to activation of GBP5 and GBP6. These GBPs then further enhance the inflammatory response via inflammasome activation. FcGR1 (CD64) is the high affinity receptor for IgG and is expressed on most myeloid cells. In humans, FcGR1 is encoded by three genes, FcGR1A, FcGR1A and FcGR1C that are highly homologous. Interaction of IgG and FcGR1 results in cellular activation, including phagocytosis, generation of reactive oxygen species, antigen-presentation, release of inflammatory cytokines, and antibody-mediated cellular cytotoxicity [54], FcGR1 expression on neutrophils has been proposed as a biomarker of infection and sepsis [55]. Neutrophils in Juvenile Idiopathic Arthritis, an inflammatory disease, express higher levels of FCGR1B compared to controls [56]. It is therefore not surprising that many signature gene sets encompassed either FcRG1A or FcRG1B. Kinase activation and phosphorylation cascades induced following immune cell activation are regulated by dual-specificity phosphatases (DUSPs) [57]. Since active TB is associated with increased inflammatory response, the presence of DUSP3 in several signature gene sets is expected. Another gene found in many signature gene sets is ANKRD22, an ankyrin repeat protein with four copies of the ankyrin motif. The motif interacts with an array of unrelated proteins to affect many cellular processes [58, 59] and it is likely that ANKRD22 expression is upregulated because of the enhanced inflammatory response in TB. Basic leucine zipper transcription factor ATF-like (*BATF*)*2*, is a transcription factor that belongs to the activator protein 1 family of transcription factors and contains the basic leucine zipper domain. *BATF2* dominance in the TB signature gene sets is consistent with its upregulation by type I IFNs [60], and by IFNγ and Mtb in macrophages [61].

The single gene biomarkers NPC2 and BATF2 were very effective in distinguishing between TB and LTBI in malnutrition. Although these single gene biomarkers are highly effective, activation of these genes are not specific to TB infection, but are associated with common inflammatory pathways (this may also be the case for some of the multi-gene “Disease” signatures). We note that NPC2 plays a key role in lysosomal cholesterol egress [62, 63] and the expression of NPC2 is directly regulated by the nuclear factor kappa B subunit 2 (NF-κB2) protein [64]. In addition, NPC2 plays a significant role in other infectious diseases, for example, upregulation of NPC2 is crucial for viral replication in Chikungunya, Zika, West Nile and Dengue infections [65]. BATF has been shown to directly control TH17 differentiation [66], and transcriptomic analysis has established that up regulation of BATF2 in HIV-specific CD8+ T cells leads to the inhibition of T cell function [67]. Thus, although these genes are sensitive biomarkers for separating TB from LTBI, they lack in specificity to TB as their expression is associated with common processes involved in host immune responses to multiple infectious agents. Thus, we would recommend using more specific, multi-gene signatures if specificity is needed for the context.

The TBSignatureProfiler was applied to samples from severely undernourished individuals with TB and LTBI in India. This analysis found that existing blood RNA signature gene sets of TB generally work in the setting of severe undernutrition, although some differences in performance do exist. Differences seen in the application of the signature gene sets may reflect the size of the gene sets (i.e., smaller gene sets may not perform as well) and/or the settings in which those data were trained. A few selected signature gene sets do not perform optimally in the setting of severe undernutrition. These findings suggest that most TB signature gene sets are robust and could work in many different settings and with different comorbidities, but some gene sets perform slightly better in different contexts. This finding has important implications in India and many high TB-burden countries.

We had hypothesized that malnutrition might modulate the transcriptional profiles in different ways and using different mechanisms than in well-nourished individuals, but this was generally not the case. Malnutrition clearly affects the immune response with effects on macrophage activity and phagocytosis, antigen presentation, and induction of the Th1 immune response among other sequelae [29]. It is plausible that these effects were not detected because the dominant immunomodulatory effect of TB that are common between well-nourished and malnourished individuals outweigh the more specific transcriptional impacts induced by changes in nutritional status. It is also likely that some of the signature gene sets themselves were developed in settings with high rates of malnutrition, so the effect of malnutrition on TB signature gene sets was incorporated. For example, Sambarey_HIV_10 signature was trained on data obtained from participants in Chennai and Bengaluru, India where malnutrition is highly prevalent. Further investigation is needed to understand the role of inflammation and immune response in the setting of malnutrition, although we show here that most existing TB signature gene sets work well in the setting of malnutrition.

Malnutrition is not the only comorbidity that is associated with TB incidence. Endemic countries have high rates of alcohol use, diabetes, HIV and other immunomodulatory conditions [68–70]. Little has been done to explore whether blood-based transcriptional TB signatures may be altered in the setting of such comorbidities. Such studies are needed before these signatures can be accepted as validated diagnostic modalities. For example, it has been shown that the Zak_RISK_16 signature has a lower AUC in the setting of HIV infection [13]. Furthermore, transcriptional profiling of individuals with diabetes and TB demonstrate activation of pathways associated with diabetes complications [24]. It is possible that signature performance in other TB-endemic settings may also be affected by genetic or Mtb strain differences. Additional work is needed to determine the impact of other common comorbidities. The TBSignatureProfiler can play an important role in facilitating future analyses in these different settings.

This work is a demonstration that existing signature gene sets can be effectively used on samples from comorbid TB contexts, although the efficacy of the gene sets may vary. While it is unlikely that these gene signatures will be used in clinical practice to distinguish pulmonary TB from LTBI controls, our work does provide the promise that existing gene sets can be used to detect TB in circumstances where existing diagnostics are less effective, e.g. distinguishing extrapulmonary, paucibacillary, and pediatric TB from controls in malnourished individuals. In addition, evaluation of the subtle differences between signature gene set performance combined with the dissection of the gene set content may provide insight on potential mechanisms specific to demographic, comorbidities, or other context-related specifics for each patient group under consideration.

We recognize that this study has several limitations. While the study has large enough sample size to determine the significance of the signature gene sets’ abilities to distinguish between TB and LTBI, the sample size was not large enough to clearly distinguish between the performance of the top-scoring gene sets. Therefore, we can only conclude that many of the gene sets work well, but we cannot determine which is the best gene set in this context. It is possible that our results do not reflect the full spectrum of gene sets in severely malnourished individuals with LTBI, as severe malnutrition may blunt the TST response; however, our previous analyses suggest this is not universally true [71]. In addition, the characteristics of the participants with TB and LTBI differed with regard to demographics (e.g. age) and risk factors (e.g. smoking and alcohol), and we do not have power to control for these differences in our analysis. While this may lead to the confounding of signature gene set strength differences between TB and LTBI, we point out that differences in demographics and co-morbidities are quite common among the TB and LTBI populations; these data represent the population dynamics of these groups. In addition, several of our signature gene sets were trained in pediatric cohorts [13, 72], but we see no difference in performance between these child/adolescent gene sets between those trained on adults.

One final limitation of our TBSignatureProfiler platform is that many existing signature gene sets were trained on different transcriptional profiling platforms (microarrays, RNA-seq) using different machine learning and predictive modeling tools. Gene set scoring methods may not perform as well with the signature gene set compared to the original platform or method—this is an area of further development for the package that is beyond the scope of this paper. However, here we evaluate existing signature gene sets across multiple scoring methods to highlight which gene signature sets of TB are the most robust across platforms and methods, and thus should work well across a variety of predictive modeling approaches and contexts. This approach may also have the benefit of reducing the likelihood of model overfitting for individual signatures trained on specific datasets.

## Conclusion

In conclusion, we have developed the TBSignatureProfiler platform that enables the application of several dozen TB signature gene sets to new datasets. The TBSignatureProfiler allows multiple scoring options and innovative graphical outputs. Using the TBSignatureProfiler, we demonstrate that severe malnutrition does not significantly alter the predictive performance of most TB gene sets. As we move toward expanded use of signature gene sets, these findings will have relevance in India and other settings with a high TB and malnutrition burden.

## Supplementary Information

**Additional file 1.** Supplementary signature list and references. This file contains the TBSignatureProfiler software doucmentation, list of signatures  and references for all the signatures in the TBSignatureProfiler.

**Additional file 2:** **Supplementary Table 1.** Output of the table_AUC() function for the ssGSEA scored signatures and single genes. **Supplementary Figure 1.** PCA plots before and after batch correction**.** (A) Illustrates principal components colored by batch which has a significant batch effect in the first principal component, and very little separation on the first two components by TB status. The two plots to the right show the ComBat-Seq corrected data colored by batch (B) and by TB status (C). These plots clearly demonstrate the reduction of batch effects and the magnification of signal due to TB status. **Supplementary Figure 2.** A heatmap and unsupervised clustering of the 500 most differentially expressed genes clearly separates the malnourished TB individuals from the malnourished LTBI. **Supplementary Figure 3.** A heatmap displaying the scaled GSVA scores for all 47 signatures (rows) for the malnourished TB and LTBI individuals (columns). The color bar at the top designates whether the sample is from an LTBI individual (red) or an individual with active TB (green). These signatures are able to separate most (all but five) of the TB samples from the LTBI samples. The pathway signature scores are largely concordant for most of the signatures. This heatmap was generated using the SignatureHeatmap() function from the TBSignatureProfiler. **Supplementary Figure 4.** A heatmap displaying the scaled PLAGE scores for all 47 signatures (rows) for the malnourished TB and LTBI individuals (columns). The color bar at the top designates whether the sample is from an LTBI individual (red) or an individual with active TB (green). These signatures are able to separate most (all but five) of the TB samples from the LTBI samples. The pathway signature scores are largely concordant for most of the signatures. This heatmap was generated using the SignatureHeatmap() function from the TBSignatureProfiler. **Supplementary Figure 5.** (A) Boxplots of the GSVA scores for each signature individually further show that some of the signatures are highly predictive of TB compared to LTBI in malnourished individuals. (B) Boxplots for the AUCs (y-axis) from bootstrapped samples for each pathway (x-axis) using the GSVA algorithm. The GSVA scores were more variable overall compared to the ssGSEA scores (Figure 2), and several of the signature AUCs had tails below 0.5 (Lee_4, Anderson_42, Thompson_RES_5, Sloot_HIV_2, Maertzdorf_OD_100, among others). **Supplementary Figure 6.** (A) Boxplots of the PLAGE scores for each signature individually further show that some of the signatures are highly predictive of TB compared to LTBI in malnourished individuals. (B) Boxplots for the AUCs (y-axis) from bootstrapped samples for each pathway (x-axis) using the PLAGE algorithm. The PLAGE scores were more variable overall compared to the ssGSEA scores (Figure 2), although a smaller number had tails around 0.5 (Lee_4, Thompson_FAIL_13, Sloot_HIV_2). **Supplementary Figure 7.** AUC plots for the individual signatures for the (A) ssGSEA scores and the (B) GSVA scores. 95% CI bands are shown for the ROC curves.

**Additional file 3:** Supplementary Methods for “Comparing Tuberculosis Gene Signatures in Malnourished Individuals using the TBSignatureProfiler”.

**Additional file 4.** Percentage of overlapping genes between the multigene TB signatures. The values are calculated as the percentage of the genes from the signature that are present in the column signature, e.g. row 45, column 3 designates that 93.8% (15/16) of the Zak_RISK_16 genes are in the Berry_393 list, whereas row 3 column 45 shows that only 5.2% of the Berry_393 are in the smaller Zak_RISK_16 list. The large number of yellow cells indicates that there is not a lot of overlap between existing signatures.

## Data Availability

The raw and processed sequencing data from this study are available in the GEO repository, under accession numbers GSE101705 and GSE152218. Furthermore, processed sequencing data and R code used for analysis and figure generation is available in the following GitHub repository: https://github.com/wevanjohnson/tbsp_malnutrition. The TBSignatureProfiler software is available through Bioconductor (https://bioconductor.org/packages/release/bioc/html/TBSignatureProfiler.html) and GitHub (https://github.com/compbiomed/TBSignatureProfiler).

## Abbreviations

TB: Tuberculosis
LTBI: Latent TB infection
BMI: Body mass index
RePORT: Regional Prospective Observational Research in TB
JIPMER: Jawaharlal Institute of Postgraduate Medical Education & Research
TST: Tuberculin skin testing
FDR: False discovery rate
AUC: Area under the curve
ROC: Reciever operating characteristic
GBPs: Guanylate-binding proteins
DUSPs: Dual-specificity phosphatases
*BATF*: Basic leucine zipper transcription factor ATF-like
CRDF: Civilian Research and Development Foundation
