# Supplementary material for: Comparing tuberculosis gene signatures in malnourished individuals using the TBSignatureProfiler
Source: BMC Infect Dis. 2021 Jan 22;21:106. doi: 10.1186/s12879-020-05598-z (PMC7821401; doi:10.1186/s12879-020-05598-z)
Supplement: Supplementary file 2 — Additional file 2: Supplementary Table 1. Output of the table_AUC() function for the ssGSEA scored signatures and single genes. Supplementary Figure 1. PCA plots before and after batch correction. (A) Illustrates principal components colored by batch which has a significant batch effect in the first principal component, and very little separation on the first two components by TB status. The two plots to the right show the ComBat-Seq corrected data colored by batch (B) and by TB status (C). These plots clearly demonstrate the reduction of batch effects and the magnification of signal due to TB status. Supplementary Figure 2. A heatmap and unsupervised clustering of the 500 most differentially expressed genes clearly separates the malnourished TB individuals from the malnourished LTBI. Supplementary Figure 3. A heatmap displaying the scaled GSVA scores for all 47 signatures (rows) for the malnourished TB and LTBI individuals (columns). The color bar at the top designates whether the sample is from an LTBI individual (red) or an individual with active TB (green). These signatures are able to separate most (all but five) of the TB samples from the LTBI samples. The pathway signature scores are largely concordant for most of the signatures. This heatmap was generated using the SignatureHeatmap() function from the TBSignatureProfiler. Supplementary Figure 4. A heatmap displaying the scaled PLAGE scores for all 47 signatures (rows) for the malnourished TB and LTBI individuals (columns). The color bar at the top designates whether the sample is from an LTBI individual (red) or an individual with active TB (green). These signatures are able to separate most (all but five) of the TB samples from the LTBI samples. The pathway signature scores are largely concordant for most of the signatures. This heatmap was generated using the SignatureHeatmap() function from the TBSignatureProfiler. Supplementary Figure 5. (A) Boxplots of the GSVA scores for each signature individually fur [file 12879_2020_5598_MOESM2_ESM.pptx]

## Slide 1
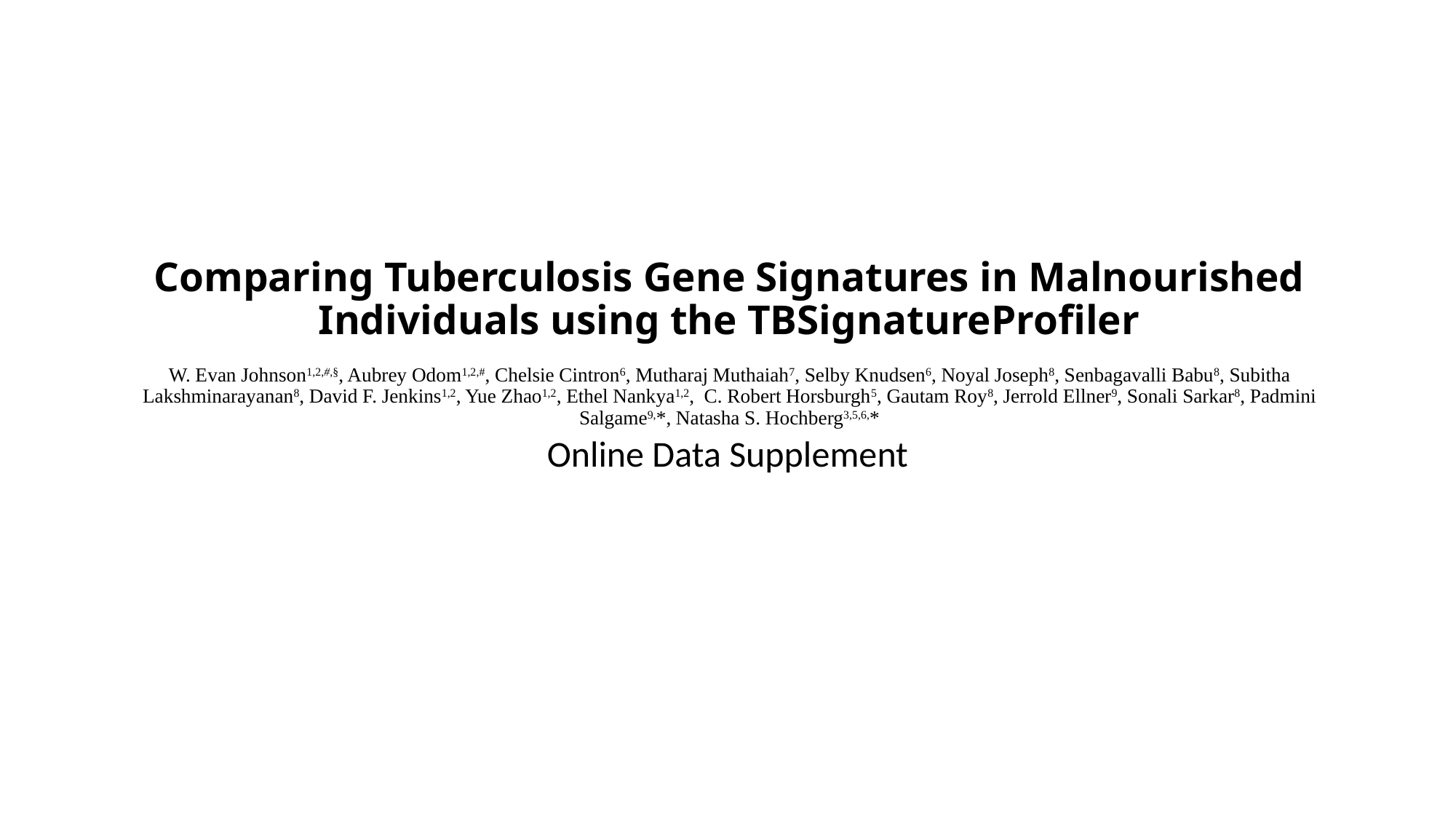

# Comparing Tuberculosis Gene Signatures in Malnourished Individuals using the TBSignatureProfiler   W. Evan Johnson1,2,#,§, Aubrey Odom1,2,#, Chelsie Cintron6, Mutharaj Muthaiah7, Selby Knudsen6, Noyal Joseph8, Senbagavalli Babu8, Subitha Lakshminarayanan8, David F. Jenkins1,2, Yue Zhao1,2, Ethel Nankya1,2, C. Robert Horsburgh5, Gautam Roy8, Jerrold Ellner9, Sonali Sarkar8, Padmini Salgame9,*, Natasha S. Hochberg3,5,6,*
Online Data Supplement

## Slide 2
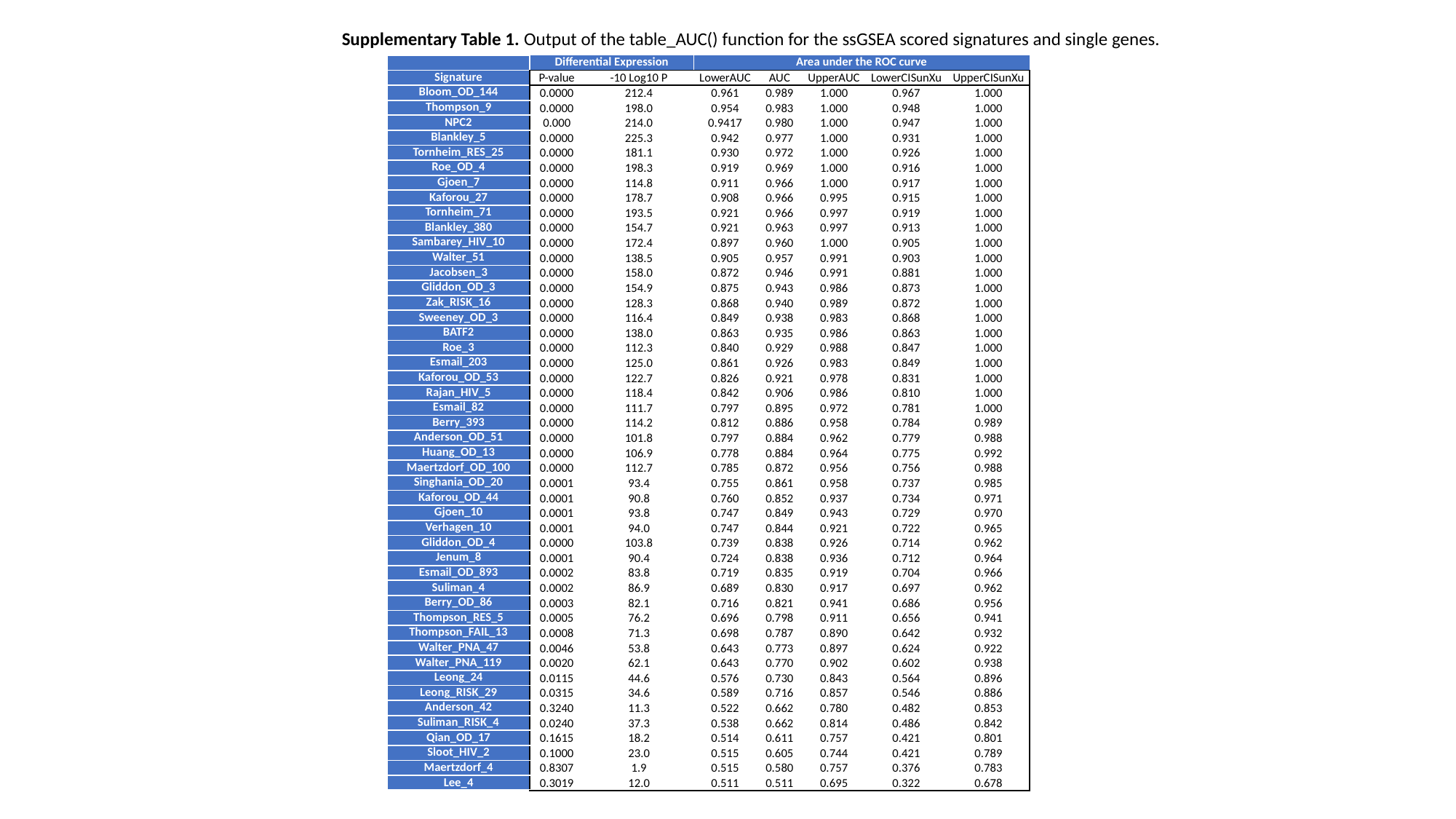

Supplementary Table 1. Output of the table_AUC() function for the ssGSEA scored signatures and single genes.
| | Differential Expression | | Area under the ROC curve | | | | |
| --- | --- | --- | --- | --- | --- | --- | --- |
| Signature | P-value | -10 Log10 P | LowerAUC | AUC | UpperAUC | LowerCISunXu | UpperCISunXu |
| Bloom\_OD\_144 | 0.0000 | 212.4 | 0.961 | 0.989 | 1.000 | 0.967 | 1.000 |
| Thompson\_9 | 0.0000 | 198.0 | 0.954 | 0.983 | 1.000 | 0.948 | 1.000 |
| NPC2 | 0.000 | 214.0 | 0.9417 | 0.980 | 1.000 | 0.947 | 1.000 |
| Blankley\_5 | 0.0000 | 225.3 | 0.942 | 0.977 | 1.000 | 0.931 | 1.000 |
| Tornheim\_RES\_25 | 0.0000 | 181.1 | 0.930 | 0.972 | 1.000 | 0.926 | 1.000 |
| Roe\_OD\_4 | 0.0000 | 198.3 | 0.919 | 0.969 | 1.000 | 0.916 | 1.000 |
| Gjoen\_7 | 0.0000 | 114.8 | 0.911 | 0.966 | 1.000 | 0.917 | 1.000 |
| Kaforou\_27 | 0.0000 | 178.7 | 0.908 | 0.966 | 0.995 | 0.915 | 1.000 |
| Tornheim\_71 | 0.0000 | 193.5 | 0.921 | 0.966 | 0.997 | 0.919 | 1.000 |
| Blankley\_380 | 0.0000 | 154.7 | 0.921 | 0.963 | 0.997 | 0.913 | 1.000 |
| Sambarey\_HIV\_10 | 0.0000 | 172.4 | 0.897 | 0.960 | 1.000 | 0.905 | 1.000 |
| Walter\_51 | 0.0000 | 138.5 | 0.905 | 0.957 | 0.991 | 0.903 | 1.000 |
| Jacobsen\_3 | 0.0000 | 158.0 | 0.872 | 0.946 | 0.991 | 0.881 | 1.000 |
| Gliddon\_OD\_3 | 0.0000 | 154.9 | 0.875 | 0.943 | 0.986 | 0.873 | 1.000 |
| Zak\_RISK\_16 | 0.0000 | 128.3 | 0.868 | 0.940 | 0.989 | 0.872 | 1.000 |
| Sweeney\_OD\_3 | 0.0000 | 116.4 | 0.849 | 0.938 | 0.983 | 0.868 | 1.000 |
| BATF2 | 0.0000 | 138.0 | 0.863 | 0.935 | 0.986 | 0.863 | 1.000 |
| Roe\_3 | 0.0000 | 112.3 | 0.840 | 0.929 | 0.988 | 0.847 | 1.000 |
| Esmail\_203 | 0.0000 | 125.0 | 0.861 | 0.926 | 0.983 | 0.849 | 1.000 |
| Kaforou\_OD\_53 | 0.0000 | 122.7 | 0.826 | 0.921 | 0.978 | 0.831 | 1.000 |
| Rajan\_HIV\_5 | 0.0000 | 118.4 | 0.842 | 0.906 | 0.986 | 0.810 | 1.000 |
| Esmail\_82 | 0.0000 | 111.7 | 0.797 | 0.895 | 0.972 | 0.781 | 1.000 |
| Berry\_393 | 0.0000 | 114.2 | 0.812 | 0.886 | 0.958 | 0.784 | 0.989 |
| Anderson\_OD\_51 | 0.0000 | 101.8 | 0.797 | 0.884 | 0.962 | 0.779 | 0.988 |
| Huang\_OD\_13 | 0.0000 | 106.9 | 0.778 | 0.884 | 0.964 | 0.775 | 0.992 |
| Maertzdorf\_OD\_100 | 0.0000 | 112.7 | 0.785 | 0.872 | 0.956 | 0.756 | 0.988 |
| Singhania\_OD\_20 | 0.0001 | 93.4 | 0.755 | 0.861 | 0.958 | 0.737 | 0.985 |
| Kaforou\_OD\_44 | 0.0001 | 90.8 | 0.760 | 0.852 | 0.937 | 0.734 | 0.971 |
| Gjoen\_10 | 0.0001 | 93.8 | 0.747 | 0.849 | 0.943 | 0.729 | 0.970 |
| Verhagen\_10 | 0.0001 | 94.0 | 0.747 | 0.844 | 0.921 | 0.722 | 0.965 |
| Gliddon\_OD\_4 | 0.0000 | 103.8 | 0.739 | 0.838 | 0.926 | 0.714 | 0.962 |
| Jenum\_8 | 0.0001 | 90.4 | 0.724 | 0.838 | 0.936 | 0.712 | 0.964 |
| Esmail\_OD\_893 | 0.0002 | 83.8 | 0.719 | 0.835 | 0.919 | 0.704 | 0.966 |
| Suliman\_4 | 0.0002 | 86.9 | 0.689 | 0.830 | 0.917 | 0.697 | 0.962 |
| Berry\_OD\_86 | 0.0003 | 82.1 | 0.716 | 0.821 | 0.941 | 0.686 | 0.956 |
| Thompson\_RES\_5 | 0.0005 | 76.2 | 0.696 | 0.798 | 0.911 | 0.656 | 0.941 |
| Thompson\_FAIL\_13 | 0.0008 | 71.3 | 0.698 | 0.787 | 0.890 | 0.642 | 0.932 |
| Walter\_PNA\_47 | 0.0046 | 53.8 | 0.643 | 0.773 | 0.897 | 0.624 | 0.922 |
| Walter\_PNA\_119 | 0.0020 | 62.1 | 0.643 | 0.770 | 0.902 | 0.602 | 0.938 |
| Leong\_24 | 0.0115 | 44.6 | 0.576 | 0.730 | 0.843 | 0.564 | 0.896 |
| Leong\_RISK\_29 | 0.0315 | 34.6 | 0.589 | 0.716 | 0.857 | 0.546 | 0.886 |
| Anderson\_42 | 0.3240 | 11.3 | 0.522 | 0.662 | 0.780 | 0.482 | 0.853 |
| Suliman\_RISK\_4 | 0.0240 | 37.3 | 0.538 | 0.662 | 0.814 | 0.486 | 0.842 |
| Qian\_OD\_17 | 0.1615 | 18.2 | 0.514 | 0.611 | 0.757 | 0.421 | 0.801 |
| Sloot\_HIV\_2 | 0.1000 | 23.0 | 0.515 | 0.605 | 0.744 | 0.421 | 0.789 |
| Maertzdorf\_4 | 0.8307 | 1.9 | 0.515 | 0.580 | 0.757 | 0.376 | 0.783 |
| Lee\_4 | 0.3019 | 12.0 | 0.511 | 0.511 | 0.695 | 0.322 | 0.678 |

## Slide 3
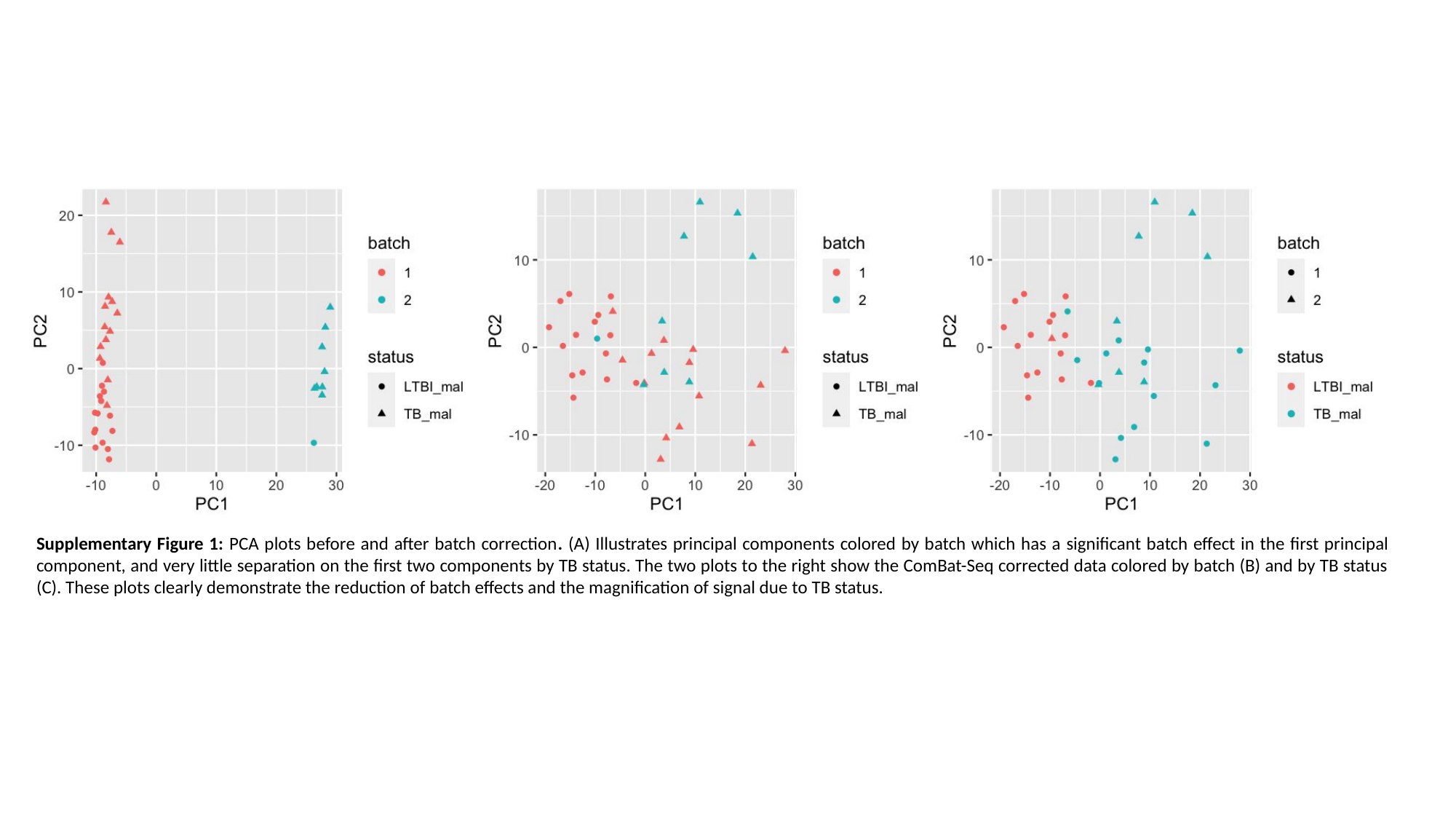

Supplementary Figure 1: PCA plots before and after batch correction. (A) Illustrates principal components colored by batch which has a significant batch effect in the first principal component, and very little separation on the first two components by TB status. The two plots to the right show the ComBat-Seq corrected data colored by batch (B) and by TB status (C). These plots clearly demonstrate the reduction of batch effects and the magnification of signal due to TB status.

## Slide 4
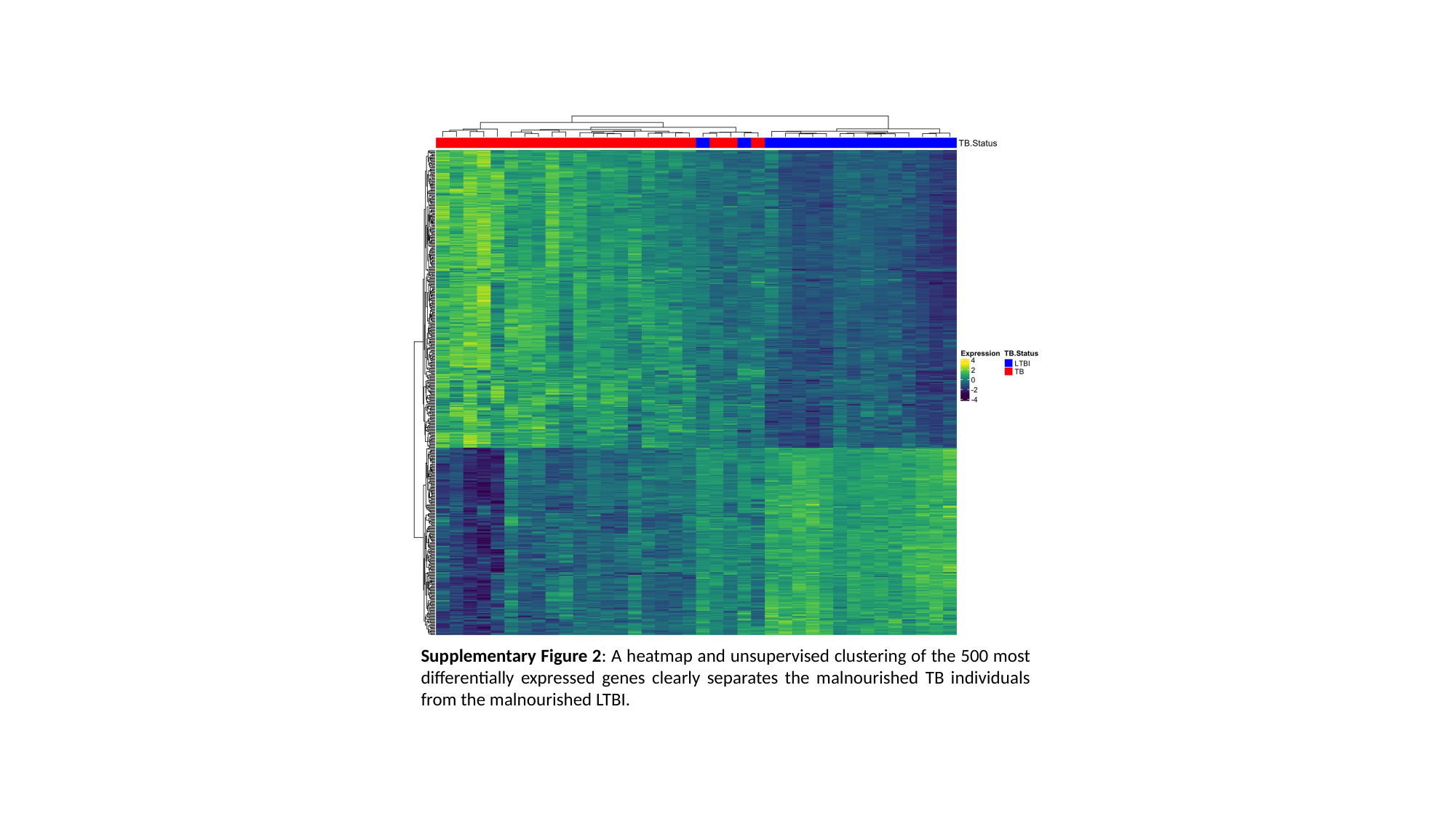

Supplementary Figure 2: A heatmap and unsupervised clustering of the 500 most differentially expressed genes clearly separates the malnourished TB individuals from the malnourished LTBI.

## Slide 5
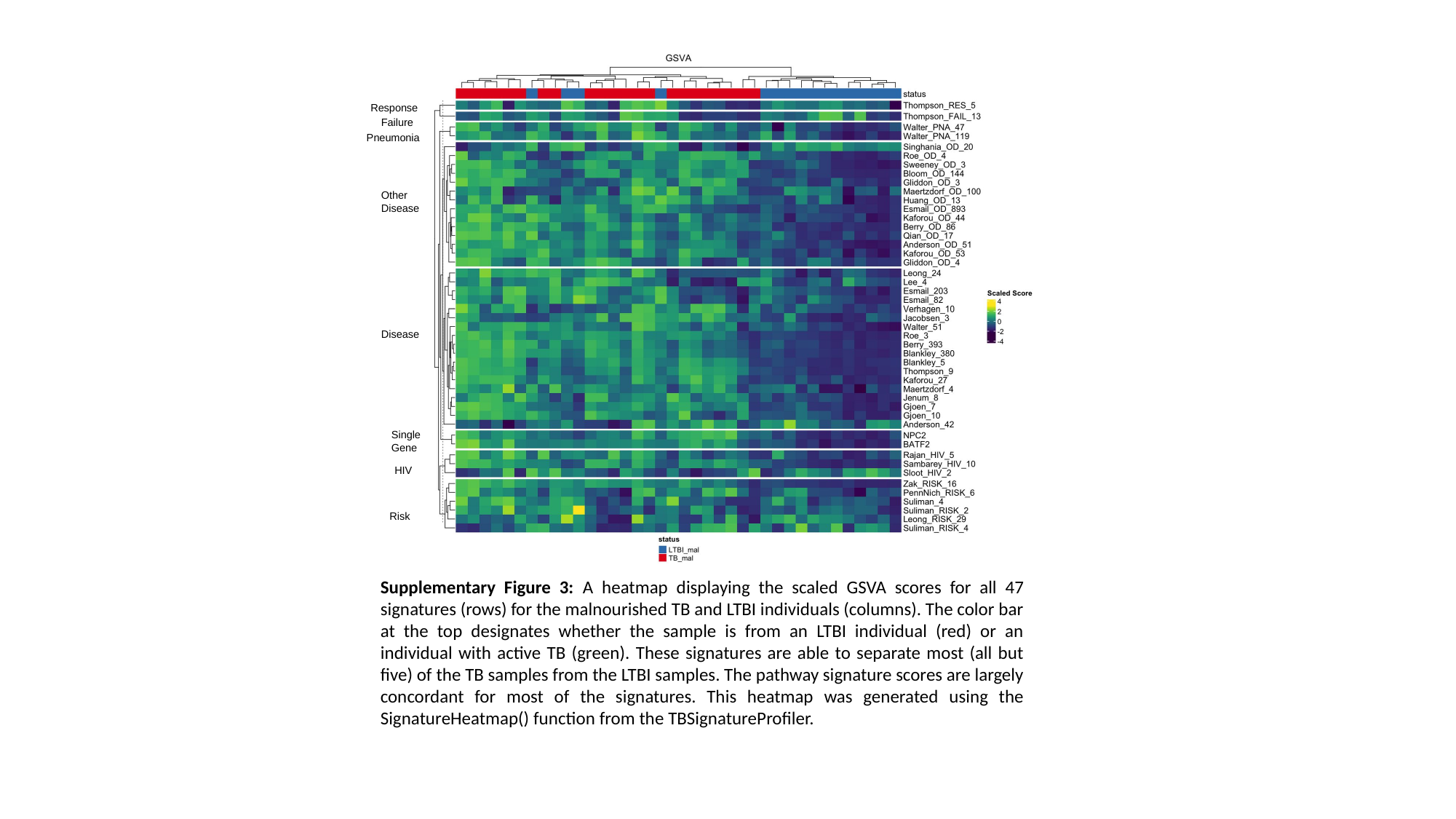

Response
Failure
Pneumonia
Other Disease
Disease
Single Gene
HIV
Risk
Supplementary Figure 3: A heatmap displaying the scaled GSVA scores for all 47 signatures (rows) for the malnourished TB and LTBI individuals (columns). The color bar at the top designates whether the sample is from an LTBI individual (red) or an individual with active TB (green). These signatures are able to separate most (all but five) of the TB samples from the LTBI samples. The pathway signature scores are largely concordant for most of the signatures. This heatmap was generated using the SignatureHeatmap() function from the TBSignatureProfiler.

## Slide 6
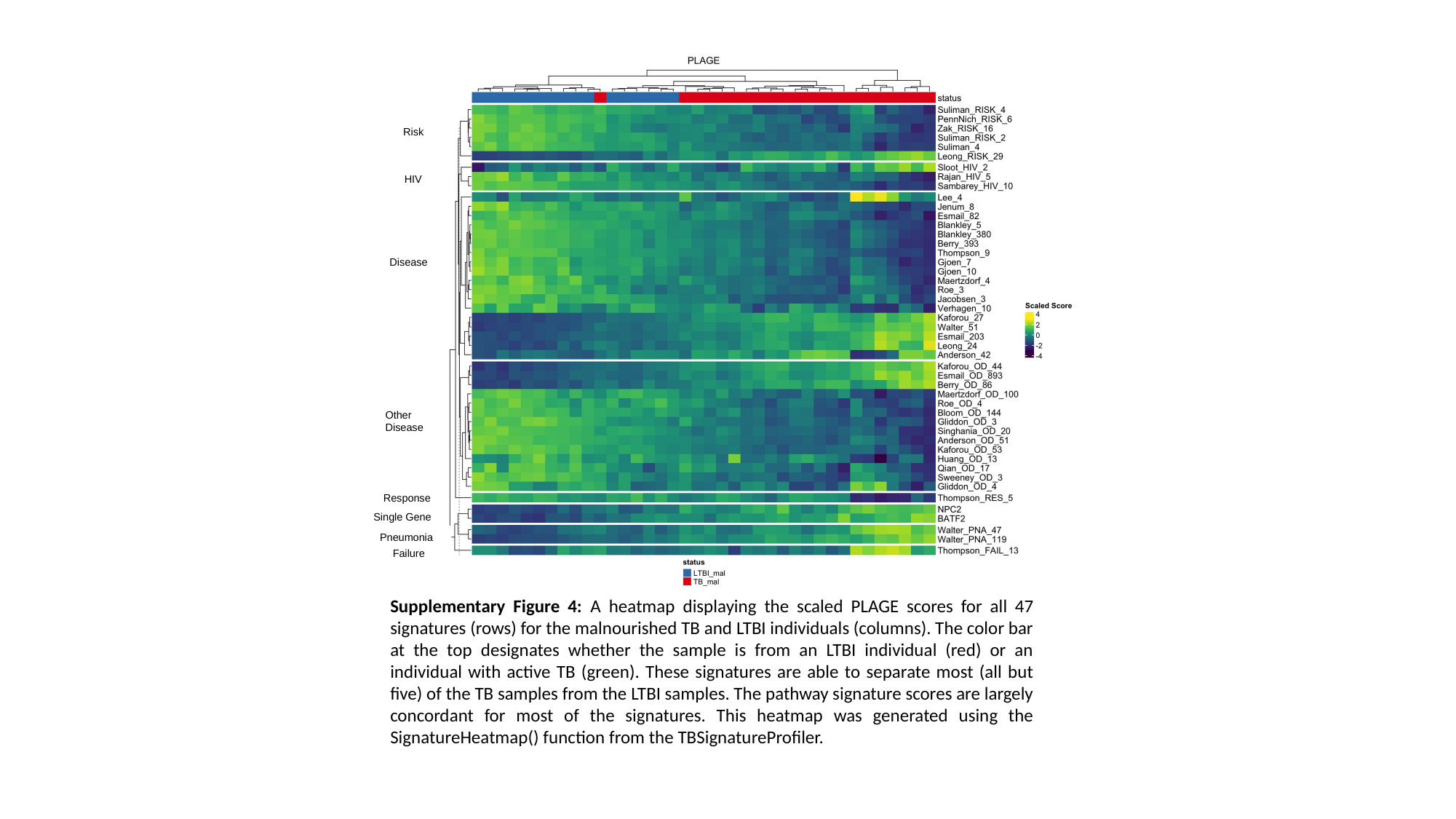

Risk
HIV
Disease
Other Disease
Response
Single Gene
Pneumonia
Failure
Supplementary Figure 4: A heatmap displaying the scaled PLAGE scores for all 47 signatures (rows) for the malnourished TB and LTBI individuals (columns). The color bar at the top designates whether the sample is from an LTBI individual (red) or an individual with active TB (green). These signatures are able to separate most (all but five) of the TB samples from the LTBI samples. The pathway signature scores are largely concordant for most of the signatures. This heatmap was generated using the SignatureHeatmap() function from the TBSignatureProfiler.

## Slide 7
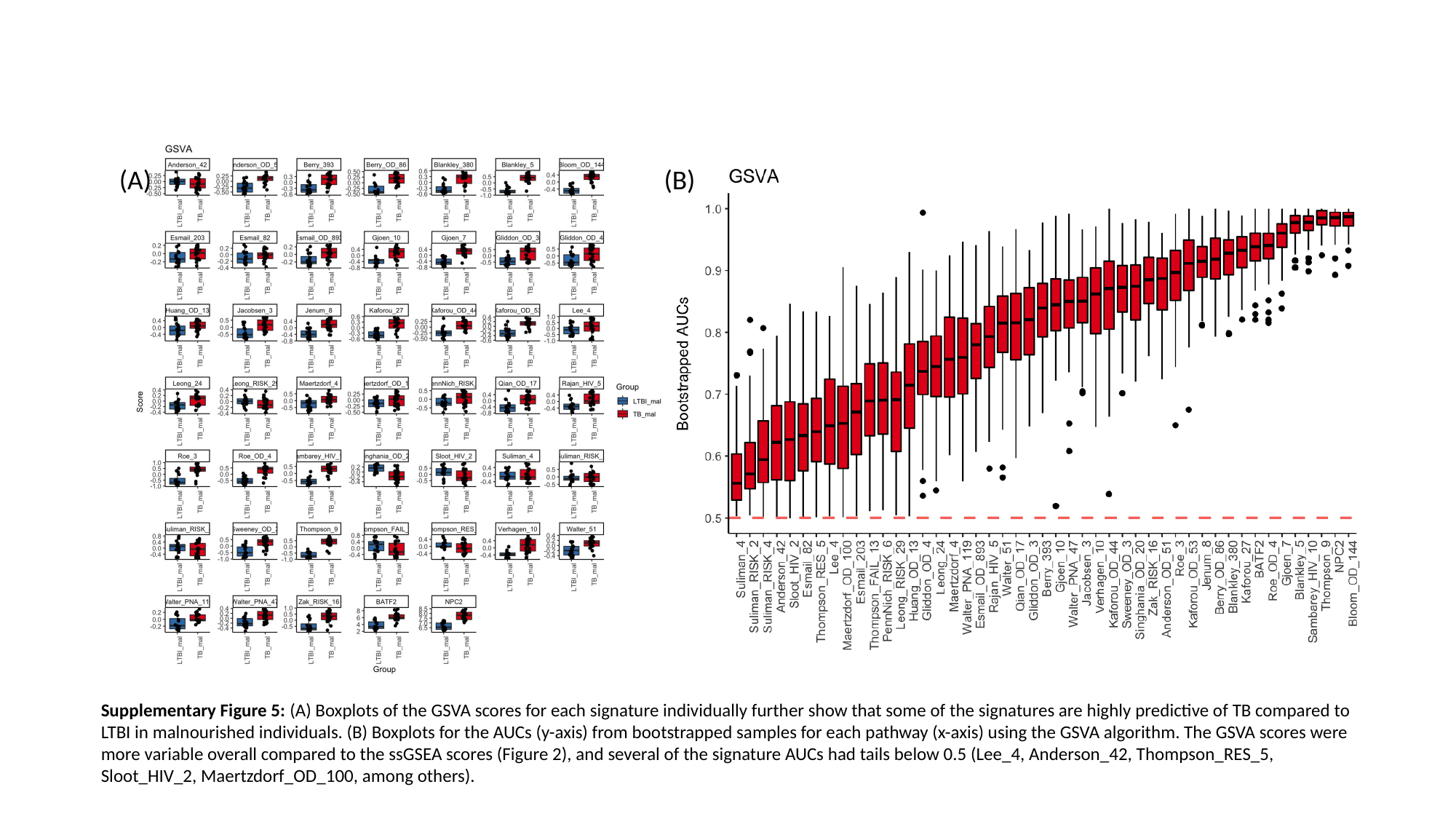

(B)
(A)
Supplementary Figure 5: (A) Boxplots of the GSVA scores for each signature individually further show that some of the signatures are highly predictive of TB compared to LTBI in malnourished individuals. (B) Boxplots for the AUCs (y-axis) from bootstrapped samples for each pathway (x-axis) using the GSVA algorithm. The GSVA scores were more variable overall compared to the ssGSEA scores (Figure 2), and several of the signature AUCs had tails below 0.5 (Lee_4, Anderson_42, Thompson_RES_5, Sloot_HIV_2, Maertzdorf_OD_100, among others).

## Slide 8
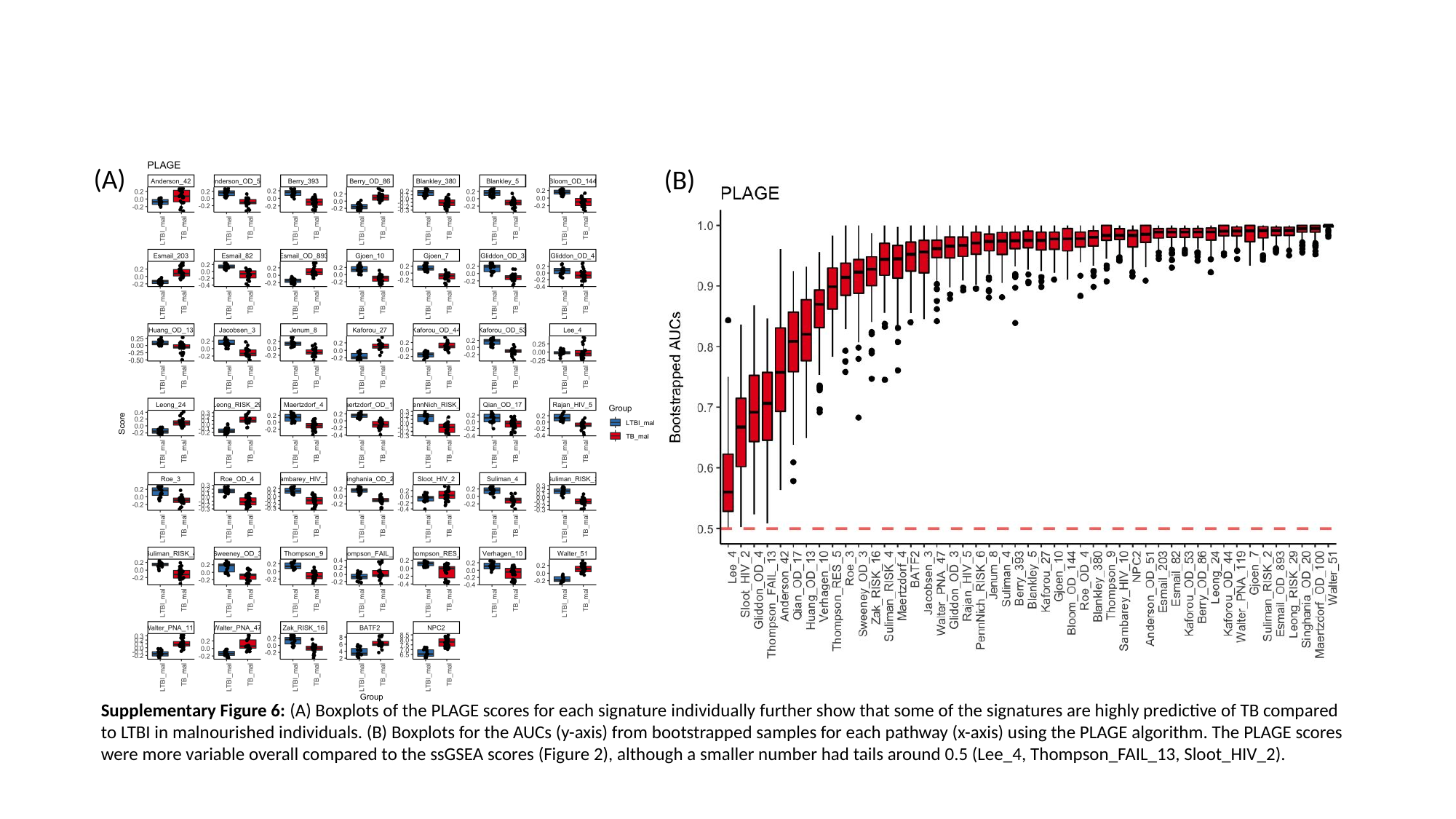

(A)
(B)
Supplementary Figure 6: (A) Boxplots of the PLAGE scores for each signature individually further show that some of the signatures are highly predictive of TB compared to LTBI in malnourished individuals. (B) Boxplots for the AUCs (y-axis) from bootstrapped samples for each pathway (x-axis) using the PLAGE algorithm. The PLAGE scores were more variable overall compared to the ssGSEA scores (Figure 2), although a smaller number had tails around 0.5 (Lee_4, Thompson_FAIL_13, Sloot_HIV_2).

## Slide 9
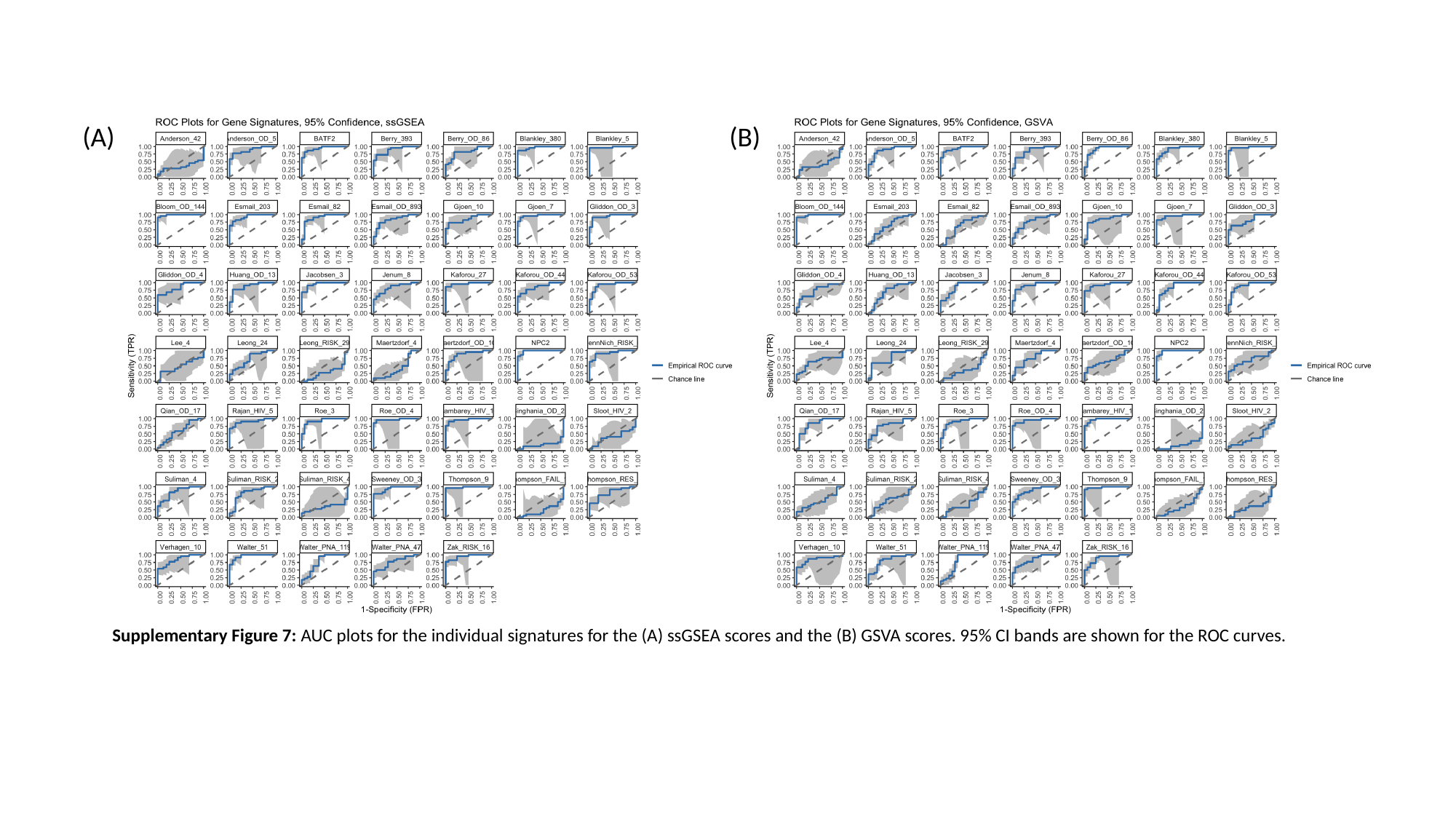

(A)
(B)
Supplementary Figure 7: AUC plots for the individual signatures for the (A) ssGSEA scores and the (B) GSVA scores. 95% CI bands are shown for the ROC curves.
