# Supplementary material for: Comparing tuberculosis gene signatures in malnourished individuals using the TBSignatureProfiler
Source: BMC Infect Dis. 2021 Jan 22;21:106. doi: 10.1186/s12879-020-05598-z (PMC7821401; doi:10.1186/s12879-020-05598-z)
Supplement: Supplementary file 3 — Additional file 3: Supplementary Methods for “Comparing Tuberculosis Gene Signatures in Malnourished Individuals using the TBSignatureProfiler”. [file 12879_2020_5598_MOESM3_ESM.docx]

**Supplementary Methods for “Comparing Tuberculosis Gene Signatures in Malnourished Individuals using the TBSignatureProfiler”**

**RNA-sequencing dataset design and generation.** We analyzed RNA-seq data from enrollment PaxGene tubes from a subset of index cases and tuberculin skin test positive (TST ≥5mm) household contacts. PaxGene tubes were sent to MedGenome (Bangalore, India) for processing. RNA was extracted from thawed samples using the PAXgene Blood RNA kit (Cat #762164, Qiagen, Hilden, Germany). Library preparation and sequencing was performed as described previously^1^. We combined two batches of data and focused on only the severely malnourished samples from the combined dataset. The first batch consisted of *n*=44 RNA-seq samples from our previously published study (GSE101705)^1^. Of these samples, only *n*=9 (*n*=8 TB, *n*=1 LTBI) were classified as severely malnourished. The second batch consisted of 48 samples, including *n*=32 severely malnourished individuals (*n*=16 TB, *n*=16 LTBI; GSE152218).

**RNA-sequencing data processing.** Raw sequencing FASTQ files were assessed for data quality using FastQC^2^. Trimmomatic^3^ was used to trim the reads (SLIDINGWINDOW:4:20 LEADING:3 TRAILING:3 MINLEN:36). Rsubread^4^, a highly efficient and accurate aligner for mapping RNA sequencing reads, was used to align reads to human genome hg38 and to determine expression counts for each gene^5^. The gene expression count matrix was filtered to remove non-expressed genes prior to differential expression analysis. We corrected for batch effects using ComBat-Seq, including TB and malnutrition status as covariates^6,7^. **Supplementary Figure 1** shows the PCA plots before and after batch correction**.** Before batch correction, samples clustered primarily by batch and not by TB status, indicating that batch-to-batch variation was highly influential. After correction, all data points, with the exception of a few outliers, congregate based on TB status regardless of batch

Principal component analysis of the raw data revealed three outlier samples (*n*=1 TB and *n*=2 LTBI) from the second batch that could not be corrected by various methods of normalization or transformation and were subsequently removed prior to downstream analysis. We were left with 23 severely malnourished TB cases and 15 severely malnourished LTBI. Differentially expressed genes (DEGs) between active TB and LTBI groups were identified using Limma and edgeR^8^. The default parameters of Limma were used, with the model design incorporating individuals' TB condition. Differential expression between TB and LTBI samples produced 6,706 differentially expressed features using an adjusted p-value (FDR) cutoff of 0.01, including 4,913 protein coding genes, 1,052 lncRNAs, 135 T cell receptive elements, 19 immunoglobulin genes, and 13 miRNAs.

**Collection of Published TB Signature Gene Sets.** We collected a set of 45 previously published gene signature gene sets that detect patients with TB, patients that are at risk of TB treatment failure, or patients that have LTBI that is likely to progress to active TB disease (**Table 2**). References for these signature gene sets are available in the **Supplementary Materials** and from the TBSignatureProfiler software documentation (?TBSignatures). These signature gene sets were derived from multiple studies, using several transcriptional profiling platforms, and using disparate predictive methods and algorithms. As such, we defined the term “signature gene set” as the collection of genes that were used in the predictive model in its particular study. We then define the “signature gene set strength” or “signature gene set score” by the single sample gene set enrichment score for that signature gene set. For presentation and consistency, signature gene sets that observe differences between LTBI and TB or a control and TB, are labeled using the first author’s last name and the number of genes in the signature (e.g., Berry_393). Signatures that focus on the presence of comorbidity with TB and another disease include an abbreviation of “other disease” (OD) or a specific disease (e.g., pneumonia as PNA, HIV) in the signature label, such as the “Gliddon_OD_3”^9^ and “Walter_PNA_47”^10^ signature gene sets. Otherwise, signature gene sets that are given a specific name in their respective publications may use some abbreviation of it in the label; for example, the 13 gene “Thompson_FAIL_13” signature gene set, and the 5 gene “Thompson_RES_5” signature gene set predict treatment failure and response to treatment^11^. Finally, if the prior publication names the signature, then we have also included it in parentheses in **Table 2** as well. All signature gene lists were compared to the gene annotations in the UCSC hg19 human reference genome. Signature genes that did not have a corresponding gene included in the hg19 gene annotation or genes that mapped to duplicate gene annotations in hg19 were removed.

**Single sample Enrichment scoring methods.** A variety of enrichment scoring methods exist to create a single score per sample; these data can then be used as a biomarker of disease in individual patients^12–17^. Single sample GSEA (ssGSEA) is an extension to the Gene Set Variation Analysis (GSEA) algorithm that provides a gene set enrichment score for each sample in a dataset given a gene list^13^. This is accomplished by ranking genes by absolute expression and calculating an enrichment score for the genes in the signature based on this ranking^13^. The GSVA calculates a similar statistic, but by first calculating an expression-level statistic using kernel estimation^17^. By contrast, Adaptive Signature Selection and Integration (ASSIGN) calculates signature activity scores using a Bayesian estimation framework to adapt signature genes to the specific context of the tested samples^12^. Pathway Level Analysis of Gene Expression (PLAGE) and the combining Z-scores method are similar in that they are both parametric, assume jointly normal distributions of gene expression profiles, and standardize each profile over the samples^17^. However, PLAGE estimates the pathway activity profiles for each gene set to be used as coefficients while the combined z-score estimates them by combining the individual gene z-scores per sample^15^. Rank-based Single Sample Scoring (singscore), the most recently developed of these methods, is a single sample scoring method which ranks genes by increasing mRNA abundance. It maintains an advantage over other methods in that it yields stable scores for individual samples by treating them independently from other samples^16^. Methods for performing ssGSEA, GSVA, PLAGE and combining Z-scores are provided in the GSVA package available in Bioconductor^18^. ASSIGN is available as a standalone package on Bioconductor^12^, as is singscore^16^.

**TBSignatureProfiler Platform, Visualization Functions, and Results.** The 45 previously published signatures of TB outcomes are included in our TBSignatureProfiler, a novel R package that allows users to quickly and easily perform single sample pathway enrichment analysis using our set of TB signature gene sets and multiple scoring methods. This workflow can then be used for profiling and visualizing these signature gene sets/pathways and plotting functions in our TBSignatureProfiler R package.

Methods for performing gene set analysis using ssGSEA, GSVA, ASSIGN, PLAGE, combining Z-scores, and singscore, in addition to boxplot and heatmap visualizations of pathway activity predictions, were included in the R package. The software utilizes the SummarizedExperiment framework to store raw expression data, annotations, and results within a single object^19^. Raw expression data is stored as a set of multiple matrices (assays) that contain identical dimensions. Along with assay data, the user can provide sample and gene annotation data to store in the colData and rowData slots, respectively^18^. Users run the runTBsigProfiler() function providing an input SummarizedExperiment object, the assay for profiling, and algorithms to use for gene set analysis. Results are per sample gene set enrichment scores stored in the colData slot of the SummarizedExperiment object and returned by the runTBsigProfiler() function. The resulting gene set enrichment results can be visualized as described above using the signatureHeatmap(), signatureBoxplot(), and signatureGeneHeatmap() functions. The compareAlgs() function profiles genetic data for a signature using multiple scoring methods and creates a heatmap comparing the scores. The R package is available on GitHub <https://github.com/compbiomed/TBSignatureProfiler>, and will be submitted to Bioconductor.

The following visualization and comparison functions are available through the TBSignatureProfiler:

*Heatmaps of multiple pathway signatures (signatureHeatmap() function):* This function uses the ComplexHeatmap R package to create an annotated heatmap of pathway signature activities^20^. Each row represents a TB signature gene set, and each column represents a sample. Pathway activity scores were scaled to highlight the differences in activity across sample; hierarchical clustering was used to identify samples that showed similar patterns of expression. Annotation information is added to the top of the heatmap as a color bar.

*Boxplots for individual pathway scores (signatureBoxplot() compareBoxplots() function):* This function generates single boxplots or an array of boxplots of signature activity using the ggplot2 R package^21^. Pathway activity scores for each signature gene set are grouped based on annotation information provided by the user.

*Heatmaps of individual pathway signatures (signatureGeneHeatmap() function):* This function creates annotated heatmaps of individual signature gene set activity^20^. Each row represents a gene, and each column represents a sample. Gene expression is scaled to highlight expression differences between samples. Annotation information is added to the top of the heatmap. Pathway activity scores are added underneath the annotation information.

*Heatmaps to compare a particular signature across scoring algorithms (compareAlgs() function):* Annotated heatmaps of specific signature gene set activity for samples are created in the same manner as the aforementioned heatmaps of individual signature gene sets. However, the heatmap rows now display the enrichment scores for each scoring algorithm given a single pathway signature gene set.

*Area under the curve (AUC) scores and p-value tables (compareBoxplots(), tableAUC(), signatureROCplot(), and signatureROCplot_CI() functions):* These functions utilize the ROCit package to calculate AUC values to evaluate signature performance^22^. These values are bootstrapped to obtain confidence interval estimates. Boxplots and receiver operator characteristic (ROC) curves of AUC values with confidence bands are produced using the ggplot2 R package^21^. HTML tables of the AUC values, bootstrapped confidence interval bounds, and p-values from 2-sample t-tests conducted on scored signature gene sets are created using the DataTables R package^23^.

**Supplementary References**

1. Leong S, Zhao Y, Joseph NM, et al. Existing blood transcriptional classifiers accurately discriminate active tuberculosis from latent infection in individuals from south India. *Tuberculosis*. 2018;109. doi:10.1016/j.tube.2018.01.002

2. Andrews S. FASTQC. https://www.bioinformatics.babraham.ac.uk/projects/fastqc/.

3. Bolger AM, Lohse M, Usadel B. Trimmomatic: a flexible trimmer for Illumina sequence data. *Bioinformatics*. 2014;30(15):2114-2120. doi:10.1093/bioinformatics/btu170

4. Rahman M, Jackson LK, Johnson WE, Li DY, Bild AH, Piccolo SR. Alternative preprocessing of RNA-Sequencing data in The Cancer Genome Atlas leads to improved analysis results. *Bioinformatics*. 2015;31(22):3666-3672. doi:10.1093/bioinformatics/btv377

5. Liao Y, Smyth GK, Shi W. The Subread aligner: Fast, accurate and scalable read mapping by seed-and-vote. *Nucleic Acids Res*. 2013;41(10). doi:10.1093/nar/gkt214

6. Johnson WE, Li C, Rabinovic A. Adjusting batch effects in microarray expression data using empirical Bayes methods. *Biostatistics*. 2007;8(1):118-127. doi:10.1093/biostatistics/kxj037

7. Zhang Y, Parmigiani G, Johnson WE. ComBat-Seq: batch effect adjustment for RNA-Seq count data. *bioRxiv*. January 2020:2020.01.13.904730. doi:10.1101/2020.01.13.904730

8. McCarthy DJ, Chen Y, Smyth GK. Differential expression analysis of multifactor RNA-Seq experiments with respect to biological variation. *Nucleic Acids Res*. 2012;40(10):4288-4297. doi:10.1093/nar/gks042

9. Gliddon HD, Kaforou M, Alikian M, et al. Identification of reduced host transcriptomic signatures for tuberculosis and digital PCR-based validation and quantification. *bioRxiv*. January 2019:583674. doi:10.1101/583674

10. Walter ND, Miller MA, Vasquez J, et al. Blood transcriptional biomarkers for active tuberculosis among patients in the United States: A case-control study with systematic cross-classifier evaluation. *J Clin Microbiol*. 2016;54(2):274-282. doi:10.1128/JCM.01990-15

11. Thompson EG, Du Y, Malherbe ST, et al. Host blood RNA signatures predict the outcome of tuberculosis treatment. *Tuberculosis (Edinb)*. 2017;107:48-58. doi:10.1016/j.tube.2017.08.004

12. Shen Y, Rahman M, Piccolo SRSRR, et al. ASSIGN: context-specific genomic profiling of multiple heterogeneous biological pathways. *Bioinformatics*. 2015;31(11):1745-1753. doi:10.1093/bioinformatics/btv031

13. Barbie DA, Tamayo P, Boehm JS, et al. Systematic RNA interference reveals that oncogenic KRAS-driven cancers require TBK1. *Nature*. 2009;462(7269):108-112. doi:10.1038/nature08460

14. Tomfohr J, Lu J, Kepler TB. Pathway level analysis of gene expression using singular value decomposition. *BMC Bioinformatics*. 2005;6(1):225. doi:10.1186/1471-2105-6-225

15. Lee E, Chuang HY, Kim JW, Ideker T, Lee D. Inferring pathway activity toward precise disease classification. *PLoS Comput Biol*. 2008;4(11). doi:10.1371/journal.pcbi.1000217

16. Foroutan M, Bhuva D, Lyu R, Horan K, Cursons J, Davis M. Single sample scoring of molecular phenotypes. *BMC Bioinformatics*. 2018;19. doi:10.1186/s12859-018-2435-4

17. Hänzelmann S, Castelo R, Guinney J. GSVA: gene set variation analysis for microarray and RNA-Seq data. *BMC Bioinformatics*. 2013;14(1):7. doi:10.1186/1471-2105-14-7

18. Huber W, Carey VJ, Gentleman R, et al. Orchestrating high-throughput genomic analysis with Bioconductor. *Nat Methods*. 2015;12(2):115-121. doi:10.1038/nmeth.3252

19. Morgan M, Obenchain V, Hester J, Pagès H. SummarizedExperiment: SummarizedExperiment container. 2019.

20. Gu Z, Eils R, Schlesner M. Complex heatmaps reveal patterns and correlations in multidimensional genomic data. *Bioinformatics*. 2016;32(18):2847-2849. doi:10.1093/bioinformatics/btw313

21. Wickham H. Getting started with qplot BT - ggplot2: Elegant Graphics for Data Analysis. In: Wickham H, ed. New York, NY: Springer New York; 2009:9-26. doi:10.1007/978-0-387-98141-3_2

22. Khan MRA, Brandenburger T. ROCit: Performance Assessment of Binary Classifier with Visualization. 2019. https://cran.r-project.org/package=ROCit.

23. Xie, Yihui, Cheng, Joe, Tan, Xianyang, Girlich M, Freedman G, Rauh J. Package ‘ DT .’ 2019.
